# Physicochemical, structural analysis of coal discards (and sewage sludge) (co)-HTC derived biochar for a sustainable carbon economy and evaluation of the liquid by-product

**DOI:** 10.1038/s41598-022-22528-y

**Published:** 2022-10-20

**Authors:** Gentil Mwengula Kahilu, Samson Bada, Jean Mulopo

**Affiliations:** 1 DSI-NRF SARChI Clean Coal Technology Research Group, School of Chemical and Metallurgical Engineering, Faculty of Engineering and the Built Environment, University of the Witwatersrand, Wits, Johannesburg, 2050 South Africa; 2 Sustainable Energy and Environment Research Group, School of Chemical Engineering, University of Witwatersrand, Wits, PO Box 3, Johannesburg, 2050 South Africa

**Keywords:** Environmental sciences, Chemistry, Engineering, Materials science

## Abstract

This study focused on the hydrothermal treatment (HTC) of coal tailings (CT) and coal slurry (CS) and the co-hydrothermal treatment (Co-HTC) of CT, CS and sewage sludge to assess the potential for increasing the carbon content of the hydrochar produced as an enabler for a sustainable carbon economy. The optimal combination methodology and response surface methodology were used to study the relationship between the important process parameters, namely temperature, pressure, residence time, the coal-to-sewage-sludge ratio, and the carbon yield of the produced hydrochar. The optimized conditions for hydrochar from coal tailing (HCT) and hydrochar from coal slurry (HCS) (150 °C, 27 bar, 95 min) increased fixed carbon from 37.31% and 53.02% to 40.31% and 57.69%, respectively, the total carbon content improved from 42.82 to 49.80% and from 61.85 to 66.90% respectively whereas the ash content of coal discards decreased from 40.32% and 24.17% to 38.3% and 20.0% when compared CT and CS respectively. Optimized Co-HTC conditions (208 °C, 22.5bars, and 360 min) for Hydrochar from the blend of coal discards and sewage sludge (HCB) increased the fixed carbon on a dry basis and the total carbon content from 38.67% and 45.64% to 58.82% and 67.0%, when compared CT and CS respectively. Carbonization yields for HCT, HCS, and HCB were, respectively, 113.58%, 102.42%, and 129.88%. HTC and Co-HTC increase the calorific value of CT and CS, to 19.33 MJ/kg, 25.79 MJ/kg, respectively. The results further show that under Co-HTC conditions, the raw biomass undergoes dehydration and decarboxylation, resulting in a decrease in hydrogen from 3.01%, 3.56%, and 3.05% to 2.87%, 2.98%, and 2.75%, and oxygen from 8.79%, 4.78, and 8.2% to 5.83%, 2.75%, and 6.00% in the resulting HCT, HCS, and HCB, respectively. HTC and Co-HTC optimal conditions increased the specific surface area of the feedstock from 6.066 m^2^/g and 6.37 m^2^/g to 11.88 m^2^/g and 14.35 m^2^/g, for CT and CS, respectively. Total pore volume rose to 0.071 cm^3^/g from 0.034 cm^3^/g, 0.048 cm^3^/g, and 0.09 cm^3^/g proving the ability of HTC to produce high-quality hydrochar from coal discards alone or in conjunction with sewage sludge as precursors for decontamination of polluted waters, soil decontamination applications, solid combustibles, energy storage, and environmental protection.

## Introduction

South Africa (SA), one of the world's leading coal producers, is largely reliant on coal to supply its energy needs^1^. According to the Department of Energy's 2001 National Coal Discard and Slurry Inventory, about 65 million tons of coal-wastes are produced each year, with the bulk of these wastes being disposed of in tailings piles and slurry dams^2^. Coal waste disposal is viewed as a serious threat to the country's environmental waste management due to the solubilization of toxic chemicals from coal waste and the possibility of spontaneous combustion^3^. Beneficiation methods such as physicochemical processes and regeneration techniques have emerged over time, however they are viewed as inefficient, unfriendly to the environment, laborious, and expensive^4^. However, sewage sludge (SS) is produced in substantial quantities by SA wastewater treatment plants^5^. The SS contains a variety of organic and inorganic pollutants that are suspected to causing illnesses (asthma, pneumonia) in people who live near disposal stockpiles^6^. Current SS management methods, such as on-site land disposal and rubbish piling, are considered unsustainable and remains a major issue^7^. As a result, innovative strategies to coal waste and SS management are deemed necessary. This study focuses on the hydrothermal carbonization (HTC) to enhance the physicochemical properties of coal tailing (CT), coal slurry (CS), and a blend of the two coal and SS in order to produce potential carbon precursors for activated carbon, and other valuable carbonaceous materials (value added products). Because it minimizes the need for an energy-intensive dewatering phase, the HTC approach is more environmentally friendly than other typical thermal processes^8^. HTC is a thermochemical process that uses hot pressurized water as a reactant and catalyst to improve the physicochemical properties of diverse raw materials^9^. The HTC products consist of a solid termed as hydrochar (HC), a liquid and a small amount of gas by-products^9^. Previous work on the HTC process assumed that CO_2_ is the predominant gas (> 95%) emitted during decarboxylation, accompanied by other gases such as CH_4_, CO, and H_2_. Under HTC conditions, the majority of the carbon and inorganic components (Ash) from the feedstocks are concentrated in the produced HC, hence reducing the quantity of CO_2_ released^9,10^. The HC synthesized is generally a stable aromatic compound with a porous structure and a high hydrophobicity^11^ level. These features inhibit the further solubilization of inorganic materials (including hazardous components) in the HC when used as adsorbent for water decontamination for instance^12^. The fuel characteristics of hydrochar produced were successfully enhanced by HTC of low carbon content coal between 150 and 270 °C (HC). Furthermore, HTC of various types of coal has indicated that the high reactivity and non-polar solvent behavior of subcritical water reduced the values of undesirable impurities such as fraction of total ash, oxygen, and sulfur while increasing the carbon content^10–12^. However, there remain a need for further experimental data to corroborate previous works on the HTC of SS or SS combined with other biomasses^13^. Additionally, previous works indicated that the carbonization and mass yields of different coal-biomass blends were extremely efficient when compared to HTC treatment of individual coal and biomass materials. The Co-HTC process provided acidic conditions that promoted the solubility of the feedstock's mineral content. As a result, when compared to HTC treatment of coal and SS individually, Co-HTC treatment of coals-sewage sludge mixture has a high likelihood of increasing feedstock carbon content^10,11,14^.

### Motivation

In coal beneficiation plants and wastewater treatment plants, the disposal of waste coal and sewage sludge, respectively, presents a significant waste management burden. Hydrothermal carbonization (HTC) is an attractive thermochemical conversion method because it can directly convert moist biomass into energy and chemical products without the need for pretreatment and provides a long-term method for reducing human-caused CO_2_ emissions by enhancing CO_2_ immobilization on a durable carbon support. Hydrochar's capacity to generate active carbon precursors for decontamination of polluted waters, soil decontamination applications, solid combustibles, energy storage, and environmental protection has led to its increasing popularity.

Despite the fact that hydrothermal conversion, hydrochar formation mechanisms, and hydrochar properties have been relatively well studied^10,13,14^, a deeper understanding of the interactions between process parameters related to the hydrothermal conversion of biomass and synthesized hydrochar structural and physicochemical proprieties for various available biomass materials is still necessary to expand hydrochar production and applications. Moreover, these correlations between the hydrothermal process conditions (temperature, heating rate, particle size, substrate concentration, catalyst addition, residence, etc.) and the physicochemical and structural properties of the hydrochar are essential in order to improve conversion. efficiency and provide reference data for industrial design and manufacturing. In addition, the liquid byproduct of hydrothermal conversion (HTC) has received little consideration, despite the necessity for a more in-depth investigation to clarify the relationship between HTC and the synthesis of hydrochar. The aforementioned provides the motivation behind paper.

## Experimental method

### Materials

**Figure 1:**
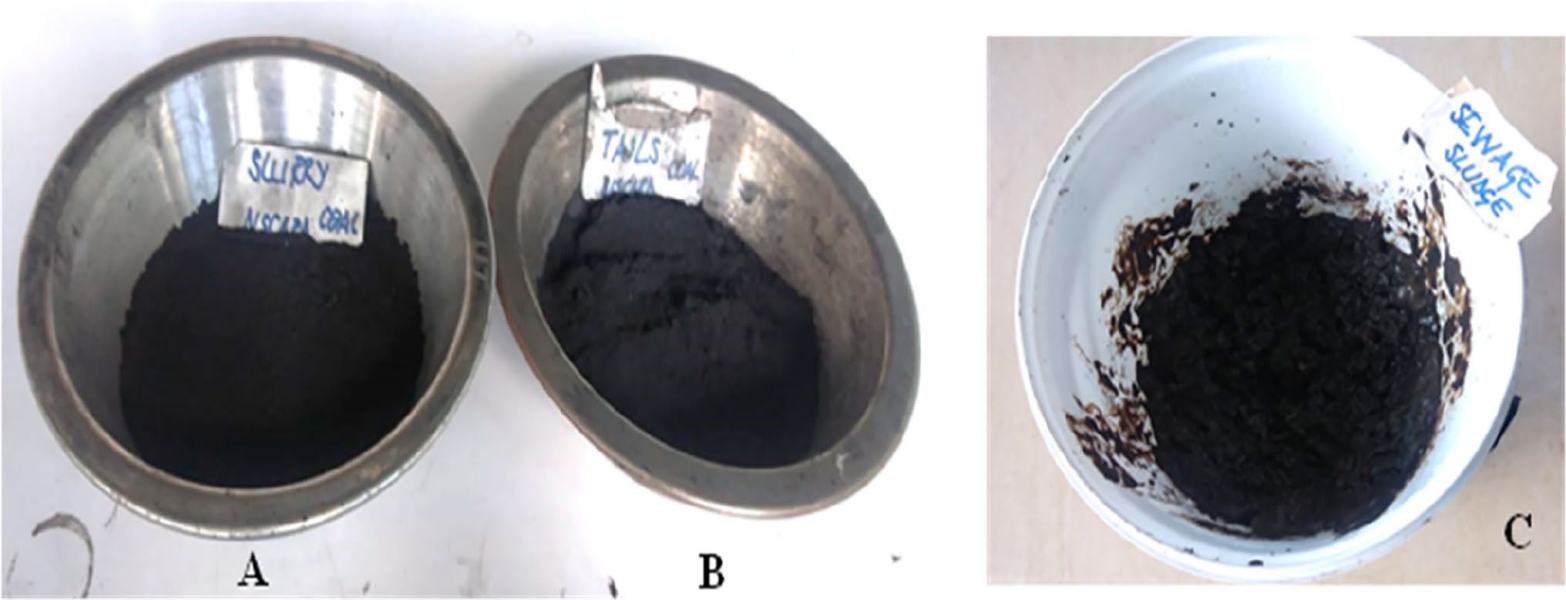
collected waste coal slurry (**A**), waste coal tailing (**B**) and sewage sludge sample (**C**).

Coal tailing (CT) and coal slurry (CS) were collected from a coal beneficiation plant in Mpumalanga, South Africa, for use in this study (Fig. 1). Before use, the samples were kept in an airtight bag in the laboratory and dried at room temperature. The dried samples were screened into various particle sizes in accordance with ASTM D5142 standards for proximal, ultimate, and total sulphur analysis. Sewage Sludge (SS) collected from a wastewater treatment plant [Ekurhuleni Water Care Company (ERWAT)] was split into two portions. The first portion was dried in a laboratory dryer at 105 °C for 24 h and crushed according to ASTM D5142 standards for physiochemical characterization, while the second portion was used for HTC tests in its collected condition.

### Methods

**Figure 2:**
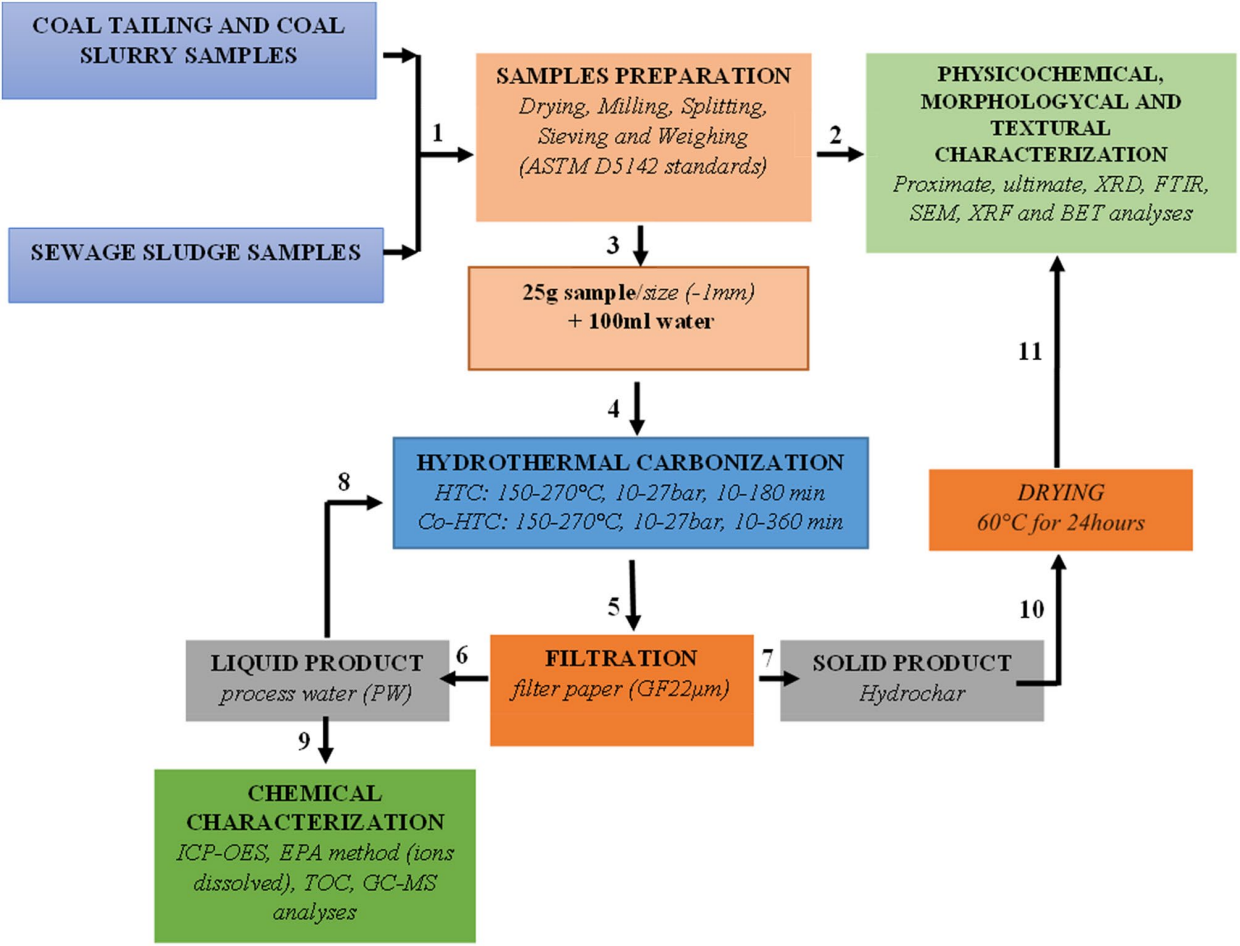
HTC and Co-HTC experimental methodology.

Hydrothermal (HTC) and co-hydrothermal (Co-HTC) studies were carried out in a high-pressure tube reactor connected to temperature module regulator and water steamer for the heat input. HTC were done on coal tailing (CT) and coal slurry (CS) separately using − 1 mm coal samples, whereas Co-HTC were performed on CD–SS mixes. 25 g of feedstock (coal or mixtures of coal and sewage sludge) was mixed with water as an input solvent in a solid to liquid ratio of 1:4. Design-Expert software (Version 13, State–Ease, Inc, Minneapolis, USA) was used to design the hydrothermal carbonization (HTC) and co-hydrothermal (HTC) experimental test runs^15^. According to the design expert matrix, the HTC and Co-HTC process parameters (temperature, pressure, residence time, and CT + CS:SS ratio) were varied. Nitrogen flow was used to keep the reactor in an inert state. After the reaction, the temperature module regulator and steamer were turned off, and the reactor was allowed to cool naturally (cooling time 180 min). To separate the liquid and solid phases, the reactor mixtures were filtered on a GF22μm filter paper disc. Hydrochar coal tailing (HCT), hydrochar coal slurry (HCS), and hydrochar blended coal and SS (HCB) are the solid products collected from HTC and Co-HTC experiments. Both solid samples were dried in an oven at 60 °C for 24 h before being prepared for characterization in accordance with ASTM D5142 standards. The design of experiment response surface methodology (DoE-RSM) technique has widely proved its accuracy in evaluating the combined interaction effects of independent (input) factors on a process^16,17^. Four hydrothermal process factors were considered in the designs: temperature, pressure, residence time, and the CD:SS (CT + CS:SS) mix ratio. The central composite design (CCD) and the custom design (CD) were used for experimental designs to determine the number of runs necessary to optimize the HTC and Co-HTC processes, respectively. HTC studies were carried out with temperature, pressure, and time changes ranging from 150 to 270 °C, 10 and 27 bar, and 10 and 180 min, respectively. The Co-HTC tests, on the other hand, were carried out with changes in residence time ranging from 10 to 360 min for the same temperature and pressure range used in HTC studies, namely 150–270 °C, 10 and 27 bar, respectively. Co-HTC experiments used a coal feedstock combination (CT + CS) of 50% CT and 50% CS. Response surface methodology (RSM), and the optimum combination methodology (OCM) were used to investigate the effects of the HTC and Co-HTC factors on the fixed carbon of the HC generated from individual CT, CS, and CD–SS mixtures, respectively. Furthermore, RSM and OCM were utilized to construct adequate models for predicting the optimum operating conditions necessary to produce high carbon content hydrochar. To assess the significance of the developed models, the analysis of variance (ANOVA) was used. The numerical optimal response approach option was then used to choose the best HTC and Co-HTC process settings, with the greatest fixed carbon (FC) content of the resultant hydrochar serving as the optimization criteria. Finally, the optimal hydrochar (HC) synthesis parameters were established and used to synthesize HCs from hydrothermal and Co-hydrothermal experiments for further physicochemical characterization. Figure 2 summarizes the methodology implemented for the experimental HTC and Co-HTC treatments.

### Chemical analyses

Proximate analysis of 1 g of − 212 µm of raw sample and produced hydrochar were carried out using the Thermogravimetric analyser equipment (Leco TGA 701) in accordance with ASTM D5142. The determination of the elemental composition (CHN) of the raw samples and produced hydrochar was done in accordance with ISO 12902 standard method using a Flash 2000 Organic Elemental Analyzer (Thermo Scientific); oxygen was calculated separately as the difference from 100% using Eq. (1).

1 $$O\%=100-\left(M+A+C+H+N+S\right)\%$$

The mass produced hydrochar mass yield was calculated using Eq. (2).

2 $$Mass \; yield \left(\%\right)=\frac{Wt\left(dried\right) \; of\, HC}{Wt \left(dried\right)\; of \; raw\;  material}$$

The carbon densification factors of the produced hydrochar were obtained using the Eq. (3)

3 $$Carbon \; desification \; factor \,{C}_{DF}=\frac{\% C\;  in\;  HC}{\% C\;  in\;  feed}$$

The Eq. (4) was used to determine the HTC and Co-HTC carbonization yields (Cy) to assess the degree of carbonization.

4 $$Carbonization \; yield\;  \left(\%\right),\,  Cy=My\frac{\% C\;  in\;  HC}{\% C\;  in\;  feed}$$

Qualitative determination of the mineral phases in the raw material and the produced hydrochar was conducted using D2 PHASER Bruker. The XRD instrument employed Cu-Kα radiation as the excitation source over a 2θ range, and a generator setting of 30 kV and 20 mA. The resulting diffractograms from the XRD analysis were matched on the Bruker D2 mineral phase database to identify the major mineral phases in the samples. The minerals phases were identified using X’PERT High-Score Plus analysis software. The FTIR analysis of raw material and produced hydrochar was conducted on a spectrometer Perkin Elmer instrument coupled with a diamond attenuated total reflectance (ATR) accessory in the wavenumber range between 4000 and 450 cm^−1^. The Scanning Electron Microscopy (SEM) of the raw material and produced hydrochar, was done using Carl Zeiss Sigma Field Scanning Electron Microscope connected to the Oxford X-act EDS detector. The analysis was carried out in this procedure according to the operational mode of the equipment. The SEM/EDS analysis settings was on 10 kV and a working distance (WD) of 7.2–8.2 mm using backscattered electrons (BSE) signal. The SEM/EDS analysis provided the surface structure and the homogeneity of the samples^18^.

The specific surface area and average pore diameter of the raw material and produced hydrochar were determined using nitrogen adsorption test at 77 K. The autosorb iQ gas sorption instrument (Quantachrome Instruments, USA) was used while the Quantachrome® ASiQwinTM software was used for data acquisition and data reduction. Information on the distribution of micropores, mesopores, and macropores was obtained from adsorption data of the N_2_ isotherm using the nonlocal density functional theory (NLDFT) method. The XRF analysis results of raw materials and produced hydrochar were obtained using the Panalytical AXIOS advanced Analyzer equipped with End-window Rh Xray tube in accordance with the Norrish Fusion technique^19^. Major elements were fused using Johnson Matthey Spectrolflux 105 at 1000 °C and raw data corrected using in-house software. Standard calibrations were made up using synthetic oxide mixtures and international standard rocks as well as in-house controls.

The produced process water (PW) from the HTC of CT, CS and Co-HTC of CB identified as LCT, LCS and LCB respectively were analyzed for potential environmental disposal assessment. The elemental inorganic and total organic matter concentration of the produced PW were determined by (ICP-OES) using an ICP-OES Agilent technologies spectrophotometer (UPMU-UTM). Prior to sample analysis, interference corrections, instrument performance, instrument detection limit, method limit and linear dynamic range were established. Samples were analyzed according to US EPA 6010 standard. The analysis was based on the ionization of a sample by hot plasma originated from an argon gas. High purity (99.99%) argon was used as plasma, auxiliary and nebulizer gas. The gas flows were kept at 15.0 l/min for plasma, 1.50 l/min for auxiliary and 0.56 l/min for nebulizer. Radio frequency (R.F) power of the plasma generator was 1.35 kW. Vertical height of the plasma was fixed at 7 mm. The charge coupled device (CCD) detector and 21 code of federal regulations (CFR) 11 version 4.1.0 software (for data acquisition) was used to analyze the samples. The chemical oxygen demand (COD) was determined using the 410.4 method of analysis^20^. The method involves using a strong oxidizing chemical, potassium dichromate Cr_2_O_7_^2−^, to oxidize the organic matter in solution for the production of carbon dioxide and water under acidic conditions.

The dissolved organic compounds of the produced PW were determined using the gas chromatograph linked to the mass spectrometer Shimadzu (GCMS-2010) The instrument was set at initial temperature of 140 °C. The initial temperature was held for five minutes then increased at 250 °C using a heating rate of 4 °C /min and maintained for 12.5 min. The samples were injected at 220 °C using the spitless mode. The analysis was performed using the following procedure: sampling time: 1.00 min; flow control mode: pressure: 100.00 kPa, total flow: 50.00 ml/min; column flow: 1.13 ml/min; linear velocity: 3.00 ml/min. The identification of the organic compounds in the PW was conducted using the NIST 14 database and compared with published mass spectra.

The total organic carbon content (TOC) of the produced PW were estimated using a chromatographic method on the Thermo-Scientific Flash elemental analyzer (TSF EA 1112) according to NF EN 15936:2013 standard. The Flash EA 1112 is based on the well-known Flash Dynamic Combustion method, which produces complete combustion of the sample within a high temperature reactor, followed by an accurate and precise determination of the elemental gases produced using a TCD thermal conductivity detector. The sample combustion temperature was 950 °C. The TOC content determination starts with the elimination of all inorganic carbons in the form of carbon dioxide by the effect of acidification of the sample with a small volume of phosphoric acid in special container. The liquid phase produced was dried at a temperature lower than 40 °C, and the special container was then closed and loaded into the auto sampler carousel for analysis. The sample quantity (100 ml) was chosen in such a way that the carbon dioxide released during combustion was in the analyzer working range. For Thermo Scientific Flash EA 1112 elemental analyzer calibration, calcium carbonate reference material was used. The value obtained after analysis is the sum of both inorganic and organic carbon in the PW or Total Carbon (TC). The following equations were used to calculate TOC of the produced PW

5 $$\mathrm{TC}=\mathrm{TOC}+\mathrm{TIC}$$

where TIC represents the total inorganic carbon of the PW obtained from the coulometer reading.

## Results and discussion

### Samples characterization

The physicochemical analysis of coals and sewage sludge samples was performed using proximate, ultimate, and total sulphur analysis to determine the quality and elemental composition of the samples. The results showed in Table 1 are reported on air dry basis.

**Table 1:** Physicochemical analysis results.

Analysis	Standards used	CT	CS	SS
**Proximate analysis (wt%, adb)**				
Moisture content	ASTDM 5142	4.17	3.94	8.12
Ash	ASTDM 5142	38.64	23.22	36.07
Volatile Matter	ASTDM 5142	21.44	21.28	47.07
Fixed Carbon	ASTDM 5142	35.75	50.98	8.75
**Ultimate analysis (wt%, adb)**				
Carbon	ISO 12, 902	42.82	61.85	29.7
Hydrogen	ISO 12, 902	3.01	3.56	4.88
Nitrogen	ISO 12, 902	1.14	1.39	4.15
Oxygen	By difference	8.79	4.78	15.22
**Total sulfur (wt%, adb)**	ISO.19579 : 2006	1.43	1.26	1.86

*CT* coal tailing, *CS* coal slurry, *SS* sewage sludge, *adb* air dried basis, *wt%* weight percentage.

The inherent moisture content of SS was found to be higher than that of CT and CS due to the nature of the sample, the process by which it was generated, and particle size distribution attributes. The CS sample has higher FC (50.98%) than the CT sample (35.75%), and SS (8.75%). Thermal treatment was required to improve the total carbon content of SS for future uses. As a result, the HTC method could improve the FC grade of the raw materials, resulting in improved porous structure properties of the produced HCs. The raw samples' ash concentration varied from 38.64 to 36.07% to 23.22% for CT, SS, and CS, respectively. The quantity of ash in the coal samples designates them as high and moderately high ash content coal (Standard South Africa ISO11760, 2005 E). This is consistent with the fact that all of the coal samples used in this study are byproducts of the coal beneficiation process.

**Figure 3:**
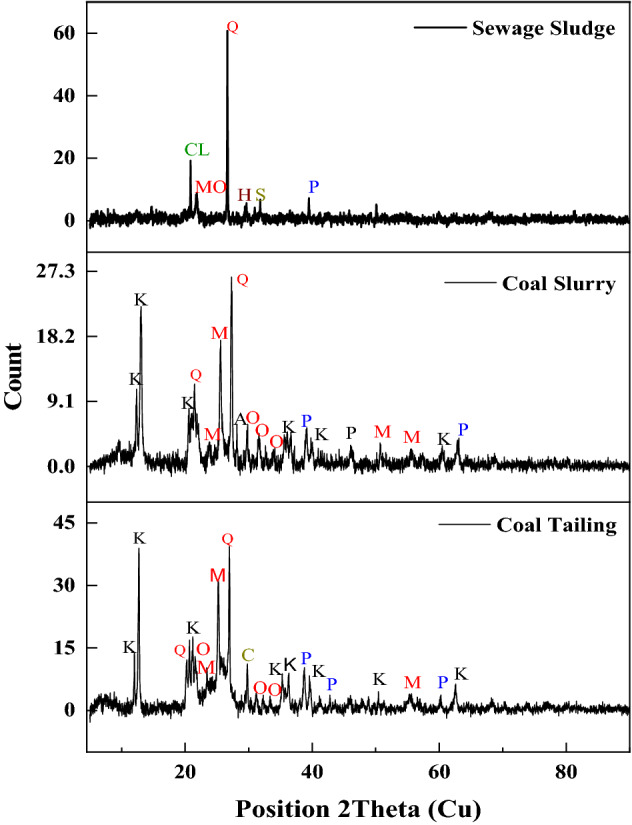
Raw samples XRD pattern. Clay Mineral phases; K (Kaolinite); Silicate mineral; M (Muscovite), AN (Anorthite), Q (Quartz), MO (Moganite), O(Orthoclase) Oxide mineral; H (Hematite), Sulphur mineral; P (Pyrite), Carbonate mineral; S (Siderite), C (Calcite); Chlorite mineral; CL ((Clinochlore).

The results from XRD analysis of raw materials are showed in Fig. 3. The observation and analysis of X-ray diffraction patterns of the coal samples revealed a predominance of crystalline mineral phases represented by intense peaks^21^. The findings indicate that mineral phases such as quartz, kaolinite, muscovite, orthoclase anorthite, hematite, chlinochlore, moganite, calcite, siderite, and pyrite dominated the composition of raw samples. A significant hump was noticed on the X-ray diffraction pattern of the CS sample compared to the CT pattern, indicating the presence of more organic components in the CS sample. The intensity of the peaks decreased from quartz, kaolinite, muscovite, orthoclase, and pyrite, reflecting the falling quantity of each phase. The CT sample shows more mineral phase peaks than the CS and SS samples. The cause might be related to the findings of proximate and petrographic examination, which revealed excessive ash and mineral matter levels in the CT, CS, and SS samples, respectively. The intensity of quartz peak in SS revealed its predominance. The results of analysis showed the pyrite sulphur mineral phase present in the two coal samples and corresponded to same position 2θ peaks. The organic hump showed a relatively less intensity in the CS sample and much less in the CS sample which corresponded to quantity of amorphous material in the samples^22^.

**Figure 4:**
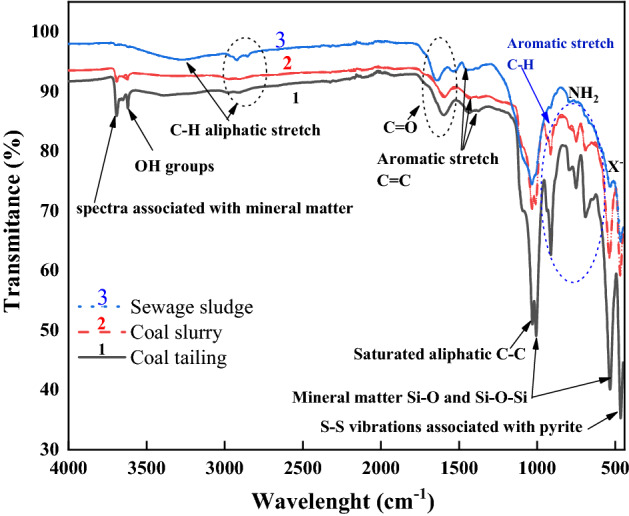
FTIR spectrum of raw samples.

The results of Fourier transform infrared analysis are presented in Fig. 4. The spectrums of raw samples present functional groups absorption peaks at corresponding wavelength. The analysis of the spectrums in absorption band between 3650 and 3250 cm^−1^ revealed the peaks at 3620 cm^−1^ for both waste coal samples showing the presence of hydrogen bond. The peaks confirm the existence of hydroxyl compound because the two spectrums contain also peaks at wavelength of 1595.25, 1029.70, 1006.19, 750.58, 691.44 cm^−1^ and 1031.34, 1008.62, 749.39 cm^−1^ for CT and CS sample respectively. The sharp intensity absorption in the absorption band range between 3670 and 3550 cm^−1^ show the presence of oxygen related group (alcohol or phenol). The absorption peaks observed at 1595.25 cm^−1^ wavelength revealed the presence of double bound carbonyl group combined with another double bound (aromatic stretch). The aromatic stretches groups reduce the intensity of carbonyl groups absorption band. The region comprised between the 1450 and 450 cm^−1^ wavelength has been found to be specific for each coal sample and is the fingerprint region of the two coal samples. Multiple band absorption groups were observed at wavelength of 1029.7, 1006.19 and 911.92 cm^−1^ on the CT spectrum and 1031.34, 1008.62 and 913.12 cm^−1^ on the CS spectrum. Vinyl-related compound corresponded to the absorption peaks at 911.92 and 913.12 cm^−1^ coal spectrums. Orto-aromatic groups was reported at wavelength of 750.58 and 749.39 cm^−1^ on the CT and CS spectrums respectively. The intense absorption peaks at wavelength of 1006.19, 465.61 cm^−1^ on the CT and 1008.69, 467.25 cm^−1^ on the CS spectrums revealed the presence of silica asymmetric stretch (Si–O–Si). The intensity of the peaks showed that the concentration of silica compound is higher in the CT sample compared to CS and SS samples. This is consistent with the results from XRD and proximate analysis. The absorption peaks at wavelength of 691.44 cm^−1^ on the CT spectrum showed the presence of monosubstituted alkynes, alkenes groups (C–H), primary and secondary amine groups (NH_2_, N–H). The absorption bands identified at 465.72 cm^−1^ wavelength in the SS sample revealed the presence mineral matter in the fingerprint region of the spectrum which was followed by an adsorption band at 1032.44 cm^−1^ showing the presence of amine, phosphate, sulfoxide, and halogenated groups. The carbonyl group with unsaturated bond has been identified at a wavelength of 1634.41 cm^−1^ on the sewage sludge spectrum. The aliphatic group identified at wavenumber 2922.63 cm^−1^ on the sewage sludge spectrum revealed the presence of long linear chains.

The results of XRF analysis are presented in Table 2. These results revealed the predominance of silicon (Si) and aluminum in the raw samples used. The highest percentages of silicon and aluminum have been found into the CT (17.021%Si and 7.14%Al) followed by the SS (11.43%Si and 5.94%Al) and CS (6.89% Si and 4.48%Al) respectively. The ferrous compounds were more indicated in the SS (5.97% Fe) compared to CT (3.31%Fe) and CS which contains a lowest percentage (1.21% Fe). Moreover, it has been observed that the CT contains high calcium (5.1% Ca) followed by SS (2.7% Ca) and CS (1.72% Ca). Those are predominant mineral’s elements present in the raw materials used. However, the analysis revealed that the samples of materials used contained also traces of some other elements such as magnesium, sodium, titanium, phosphorus, chromium manganese and nickel as shown in Table 2.

**Table 2:** XRF results of raw materials.

Element composition	CT	CS	SS
SiO_2_	36.42	14.76	24.46
Al_2_O_3_	13.50	8.48	11.23
CaO	7.14	2.41	3.79
Fe_2_O_3_	4.74	1.81	8.53
MgO	0.77	0.39	1.92
TiO_2_	0.67	0.56	0.76
K_2_O	0.52	0.23	1.03
P_2_O_5_	0.33	0.35	9.86
Na_2_O	0.10	0.08	0.78
MnO	0.03	0.02	0.21
Cr_2_O_3_	0.00	0.00	0.13
NiO	0.00	0.00	0.04
LOI	35.22	70.21	39.78
Total	99.44	99.30	99.52

*CT* coal tailing, *CS* coal slurry, *SS* sewage sludge.

The XRF results confirmed the results of precedents analysis which revealed the high content of mineral (ash) in the CT followed by SS and CS respectively. Therefore, to reduce the percentage of mineral content in the raw material, the HTC process was used to dissolve a fraction of elements via hydrolysis for the increase of carbon content.

### HTC process results

Tables 3 and 4 show the FC, A, and VM of the HC synthesized from HTC of CT and CS with a solid/liquid ratio of 1/4. The results suggest that the reaction temperature, and pressure had the greatest impact on the HTC experimental procedure, which is consistent with other works^22,23^. However, raising the temperature from 150 to 270 °C lowered the FC content of the synthesized HC i.e. after 10 min of reaction, the greatest FC grades (40.37% and 57.69%) were achieved at 150 °C with a matching pressure of 10 bar. Additionally, the SS and CD samples were mixed to produce an HC with high carbon content and porous structure. The goal was to assess the combination of these three forms of waste materials in a predetermined proportion to enhance the hydrochar synthesised. Though, some researchers have undertaken studies on the HTC of SS^24,25^, none of them have documented systematically the physicochemical properties of the products resulting from the blending of SS and CD in the HTC process. Preliminary results in Table 5 indicate that using SS in combination with CD enhance the hydrochar synthesised as the FC content of the HCs synthesised increases. This may be due to the presence of acid functional groups in the SS sample as indicated in Fig. 2. These acid functional groups i.e., phosphate, amine, and sulfoxide play an important role in the dissolving of mineral materials in coal samples during the HTC process. This is congruent with other findings that indicated that the presence of acid promotes HC synthesis during the HTC process by altering the proportionality of produced phases^26^. As a result, the carbon content of the generated HC rises, which improves its physical properties while decreasing the atomic ratios of O/C and H/C^14^. Table 5 show the influence of temperature, pressure, and residence time on the ash content, fixed carbon, and volatile matter of the hydrochar (HCs) synthesised in this study using coal discards (CD) and sewage sludge (SS).

**Table 3:** Physicochemical analysis results of produced HC from coal tailing (CT).

Run	Factors			Responses				
	A	B	C	R1	R2	R3	R4	R5
	Temperature (°C)	Pressure (bar)	Time (min)	A* (%)	FC* (%)	VM* (%)	Mass yield (%)	Carbon yield (%)
Raw sample	–	–	–	**40.32**	**37.31**	**22.37**	**100**	**100**
1	150	10	180	38.92	39.27	21.81	83.56	87.95
2	150	27	10	39.11	39.83	21.06	83.51	89.15
3	150	18.5	95	**38.36**	**40.37**	**21.27**	**82.67**	**89.44**
4	150	10	10	39	39.38	21.62	82.55	87.13
5	150	27	180	38.16	40	21.84	78.66	84.33
6	210	18.5	95	40.74	37.55	21.71	83.63	84.17
7	210	18.5	95	40.32	37.53	22.15	83.22	83.71
8	210	18.5	10	41.39	37.45	21.16	81.73	82.04
9	210	18.5	95	40.28	37.57	22.15	81.76	82.33
10	210	18.5	95	40.31	37.54	22.15	82.03	82.53
11	210	18.5	95	40.37	37.48	22.15	81.99	82.36
12	210	18.5	180	40.89	37.68	21.43	81.36	82.16
13	210	18.5	95	40.25	37.6	22.15	81.78	82.41
14	210	27	95	40.20	37.65	22.15	81.26	82.00
15	210	10	95	40.16	38.25	21.59	81.50	83.55
16	270	10	180	41.28	37.19	21.53	80.67	80.41
17	270	27	180	40.22	37.44	22.34	77.31	77.58
18	270	18.5	95	41.26	37.38	21.36	77.21	77.35
19	270	10	10	38.13	37.31	24.56	78.39	78.39
20	270	27	10	40.41	37.57	22.02	78.24	78.79

Significant values are in bold.

*A* ash content, *FC* fixed carbon, *VM* volatile matter.

*Dried basis (moisture free). Solid/liquid ratio: 1/4.

**Table 4:** Physicochemical analysis results of produced HC from coal slurry (CS) .

Run	Factors			Responses				
	A	B	C	R1	R2	R3	R4	R5
	Temperature (°C)	Pressure (bar)	Time (min)	A* (%)	FC* (%)	VM* (%)	Mass yield (%)	Carbon yield (%)
Raw sample	–	–	–	**24.17**	**53.02**	**22.69**	**100**	**100**
1	150	10	180	23.12	55.72	22.16	82.45	86.64
2	150	27	10	21.53	57.08	21.39	89.91	96.79
3	150	18.5	95	**20.12**	**57.69**	**22.19**	**86.74**	**94.38**
4	150	10	10	22.17	55.87	21.96	85.65	90.25
5	150	27	180	22.65	55.75	21.6	85.80	90.21
6	210	18.5	95	25.67	52.27	22.06	84.66	83.46
7	210	18.5	95	25.2	52.34	22.46	84.91	83.82
8	210	18.5	10	25.57	52.96	21.47	84.86	84.77
9	210	18.5	95	25.33	52.21	22.46	86.08	84.77
10	210	18.5	95	25.27	52.27	22.46	84.44	83.25
11	210	18.5	95	25.96	52.29	21.75	84.93	83.76
12	210	18.5	180	25.24	52.3	22.46	87.28	86.09
13	210	18.5	95	26.19	51.35	22.46	86.87	84.13
14	210	27	95	21.27	56.27	22.46	84.96	90.17
15	210	10	95	23.4	54.68	21.92	83.55	86.17
16	270	10	180	28.29	48.85	22.86	82.26	75.79
17	270	27	180	27.26	50.06	22.68	84.90	80.16
18	270	18.5	95	28.19	50.12	21.69	85.10	80.45
19	270	10	10	27.61	49.44	22.95	82.45	76.88
20	270	27	10	26.81	50.83	22.36	83.57	80.12

Significant values are in bold.

*A* ash content, *FC* fixed carbon, *VM* volatile matter.

*Dried basis (moisture free). Solid/liquid ratio: ¼.

**Table 5:** Physicochemical results of HC from mixture of CD and SS.

Run	Mixture components		Factors			Responses				
	A: 0.5(CT + CS) (g)	B: SS (g)	C	D	E	R1	R2	R3	R4	R5
			Temperature (°C)	Pressure (bar)	Time (min)	A* (%)	FC* (%)	VM* (%)	Mass yield (%)	Carbon yield (%)
CT	–	–	–	–	–	**40.32**	**37.31**	**22.37**	–	
CS	–	–	–	–	–	**24.29**	**53.02**	**22.69**	–	
SS	–	–	–	–	–	**39.25**	**9.52**	**51.23**	–	
CB	–	–	–	–	–	**38.8**	**38.67**	**22.53**	100	100
1	12.24	12.76	150	10	135.78	33.31	39.21	27.48	66.39	67.31
2	12.32	12.68	150	20.54	360	31.13	44.93	23.94	66.66	77.45
3	24.37	0.63	150	10	10	27.73	48.99	23.28	67.77	85.85
4	24.37	0.63	150	27	360	33.32	45.63	21.05	71.83	84.75
5	0	25	150	16.29	10	41.59	13.12	45.28	54.40	18.46
6	12.47	12.53	150	27	10	29.68	43.54	26.78	68.85	77.52
7	12.24	12.76	150	10	135.78	31.46	43.43	25.11	67.56	75.87
8	0.63	24.37	150	10	360	40.91	17.43	41.66	51.56	23.24
9	25	0	150	27	10	28.11	50.09	21.80	73.06	94.63
10	0	25	150	27	229.05	46.97	12.18	40.84	53.31	16.79
11	0	25	150	18.22	220.08	42.54	17.48	39.98	54.43	24.60
12	25	0	152.4	17.65	198.56	27.28	50.86	21.86	71.59	94.15
13	12.28	12.72	183.6	14.505	264.93	28.53	48.32	23.15	59.56	74.43
14	25	0	194.4	10	360	29.29	46.96	23.75	70.38	85.47
15	12.36	12.64	203.4	26.74	200.35	30.76	47.03	22.21	59.26	72.08
16	12.35	12.65	203.4	26.74	200.35	27.81	48.38	23.81	57.47	71.90
17	12.54	12.46	204	17.65	20	30.52	44.12	25.36	56.41	64.36
18	0	25	204	19.35	354.61	59.47	8.98	31.55	71.10	16.51
19	12.54	12.46	204	17.65	20	31.03	43.22	25.74	59.35	66.34
20	0	25	204	10.34	162.68	58.72	8.59	32.69	52.08	11.57
21	2.50	22.50	206.4	19.69	159.1	44.21	30.29	25.50	48.96	38.35
22	20.22	4.78	208.10	22.28	360	**19.71**	**58.82**	**21.46**	**65.38**	**99.45**
23	25	0	216	17.65	30	29.06	47.73	23.22	72.76	89.80
24	25	0	216.6	26.745	198.56	26.72	51.59	21.69	71.34	95.18
25	25	0	222.6	15.95	198.56	26.51	51.77	21.71	67.80	90.77
26	0	25	224.4	27	10	52.14	14.02	33.85	48.26	17.50
27	12.92	12.08	225.6	10	360	31.65	46.84	21.51	53.40	64.69
28	0	25	267	19.435	162.68	30.39	48.17	21.45	38.26	47.66
29	12.25	12.75	267	17.65	198.56	32.20	46.94	20.86	50.01	60.70
30	12.25	12.75	267	17.65	198.56	31.13	46.49	22.38	48.72	58.57
31	12.25	12.75	267	17.65	198.56	33.75	45.12	21.13	49.41	57.65
32	1.12	23.88	270	10	360	62.17	20.02	17.81	33.90	17.55
33	12.68	12.32	270	27	360	32.44	44.51	23.05	47.90	55.14
34	12.69	12.31	270	10	10	29.27	45.27	25.47	46.73	54.71
35	0	25	270	10	10	51.13	14.23	34.64	36.57	13.46
36	12.37	12.63	270	27	10	34.17	41.19	24.64	49.38	52.59
37	25	0	270	10	135.78	29.03	49.24	21.73	69.35	88.30
38	25	0	270	27	10	29.72	47.82	22.46	72.16	89.23
39	25	0	270	20.625	360	35.87	42.37	21.75	71.16	77.97
40	0	25	270	27	360	30.29	48.06	21.65	33.75	41.94

Significant values are in bold.

*HC* hydrochar, *SS* sewage sludge, *CT* coal tailing, *CS* coal slurry, *min* minutes, *°C* degrees celsius, *A* ash content, *FC* fixed carbon, *VM* volatile matter. Solid/liquid ratio: 1/4.

*Dry basis (moisture free).

Optimal HTC parameters were found considering the physicochemical data of the HC produced i.e., the optimized HC should have the maximum fixed carbon content and the lowest ash content and volatile substances. Surface response approach was used to develop linear and quadratic models.

The statistical parameters were evaluated using the analysis of variance (ANOVA) on the HTC and Co-HTC experimental results obtained from proximate analysis presented in Tables 3, 4 and 5. The regression models (Eqs. 5–7) obtained for HTC and Co-HTC using RSM and CD were used to evaluate the influence of HTC and Co-HTC factors on the FC of the produced hydrochar (HCs). In addition, these regression models were tested using the probability (*p* value) and Fischer test values (F-value) for the obtained responses. All models were found statistically significant due to high F-value and lower *p* value^16,27^. The FC models for HCT and HCS were observed as a good fit, (R^2^ = 0.9517, R^2^ = 0.8430 respectively at *p* value < 0.0001) and are represented by Eqs. (5) and (6) respectively. In addition, FC model of produced HCB was established as a good fit, (R^2^ 0.8434 at *p* value < 0.0001) and is represented by Eq. (7).

5 $$HCT \; Fixed\;  Carbon=37.66-1.20A+0.1090B+0.0040C-0.0837AB-0.0388AC+0.0337BC+1.04{A}^{2}+0.113{B}^{2}-0.2714{C}^{2}$$

6 $$HCS \; Fixed\;  Carbon=53.01-3.29A+0.5520B-0.3410C$$

7 $$HCB \; Fixed\;  Carbon=30.46A+45.36B+55.13AB+9.11AC+6.58AD+13.69AE-0.6847BC-0.1279BD-4.28BE-15.83ABC-10.52ABD+14.57AB$$

**Figure 5:**
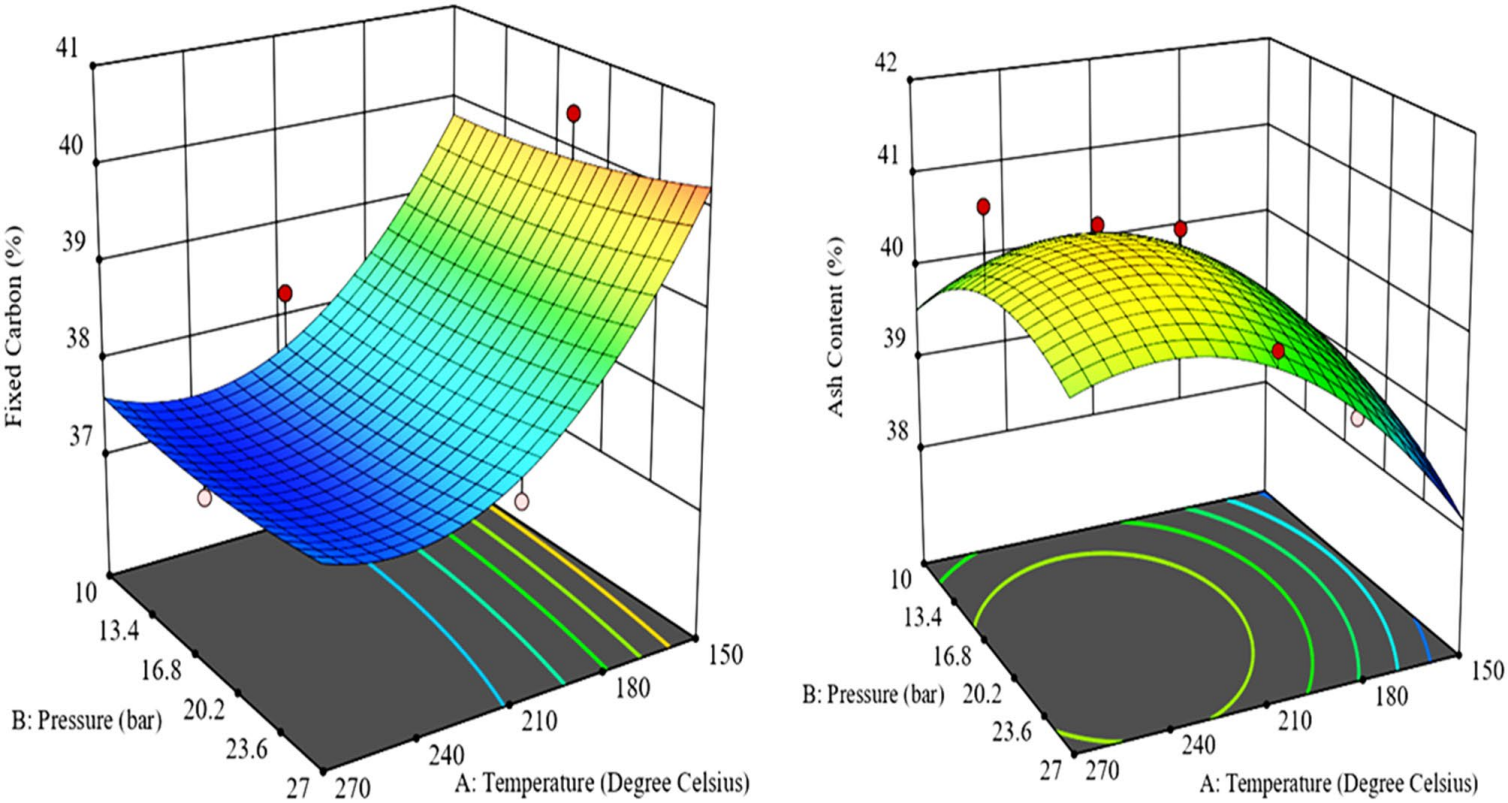
Surface plot showing the influence of temperature and pressure on fixed carbon (left) and Ash content (right) of Coal tailing’s HC.

**Figure 6:**
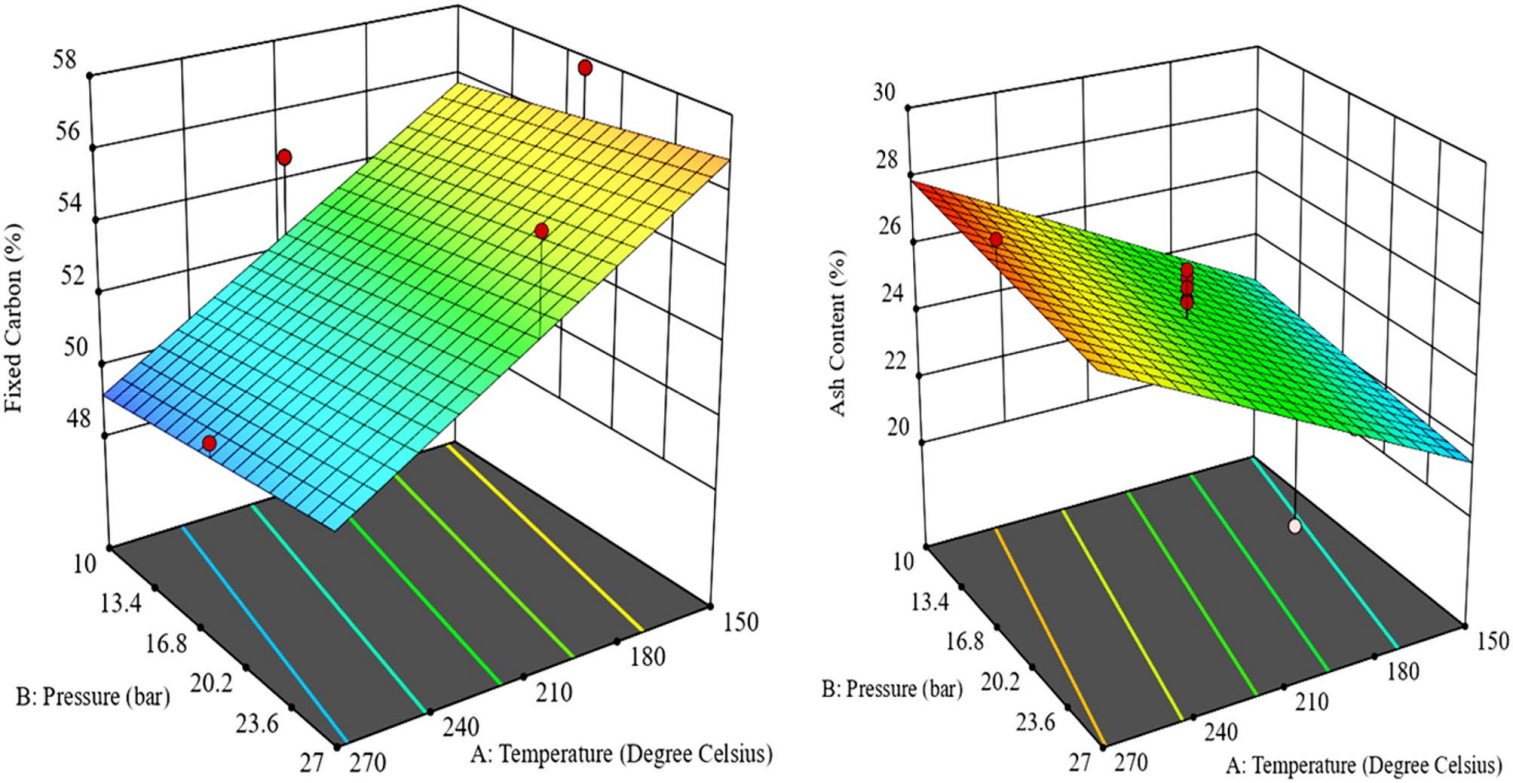
Surface plot showing the influence of temperature and pressure on fixed carbon (left) and Ash content (right) of Coal slurry’s HC.

**Figure 7:**
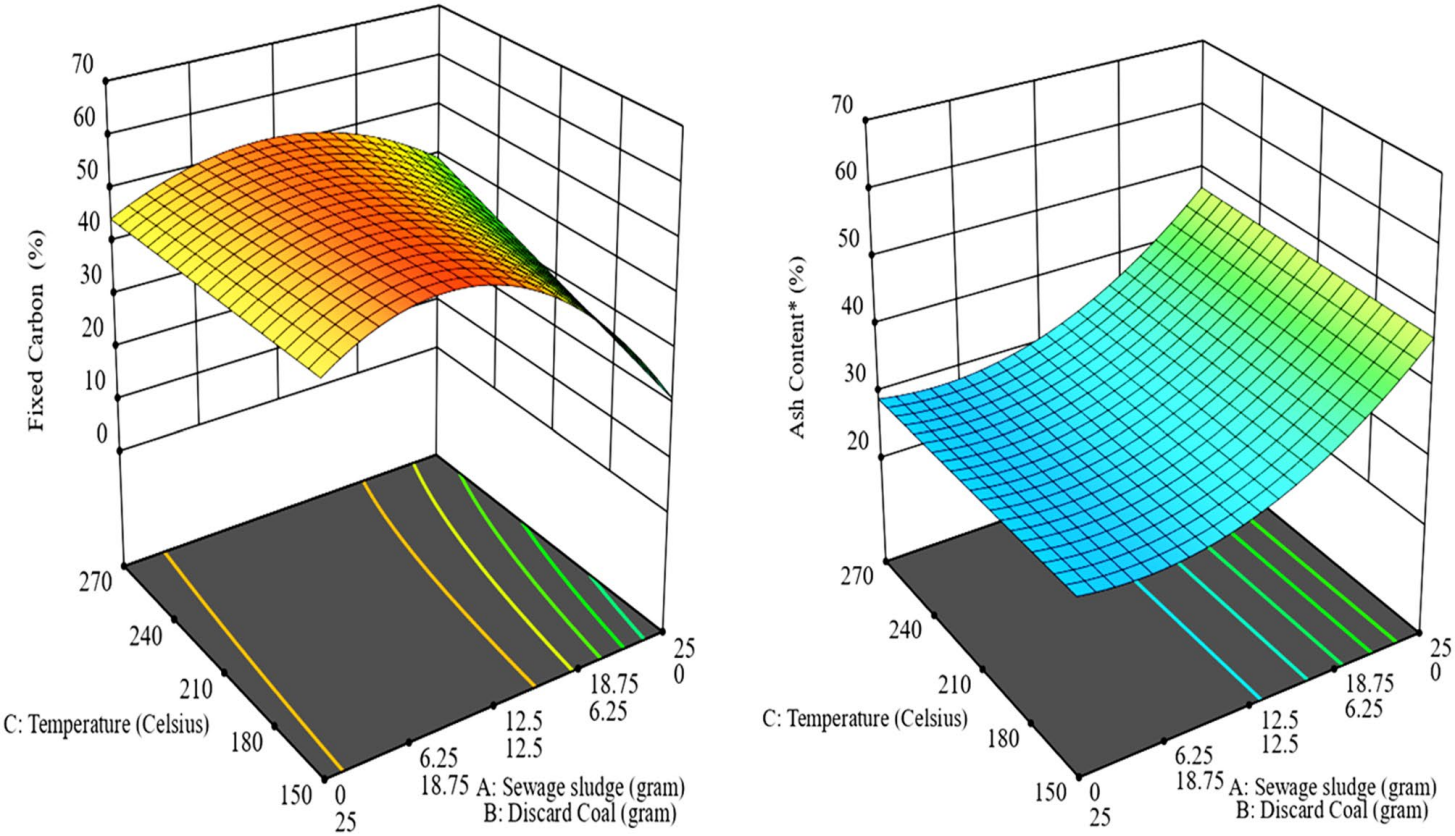
Surface plot showing the influence of temperature and pressure on fixed carbon (left) and Ash content (right) of the mixture of Sewage sludge and Coals.

The negative coefficient of the input variables indicates its antagonistic effect of on the FC value of the produced HC. The synergetic effect of the output responses was revealed by the positive coefficients within the equations^28^. The effect of operating conditions on the FC of the produced hydrochar (HC) was illustrated by the 3D plot of the response surface (Figs. 5, 6 and 7).

### Optimum operating conditions

The analysis and evaluation of obtained data were completed by the optimization of the input and responses factors (Table 9). The objective was to evaluate the input factors for the maximization of FC for the produced HCs. The input factors ranged from 150 °C, 10 bar and 10 min as lower limits to 270 °C and 27 bar, 180 min as upper limits of yielded optimization solutions for HTC process. On the other hand, the Co-HTC yielded optimization solutions were determined using input factors ranged from 150 °C, 10 bar, 10 min and 0:25 g (CT + CS:SS) as lower limits and 270 °C, 27 bar, 360 min and 25:0 g (CT + CS:SS) as upper limits. Using the desirability function, the conditions that provided the maximum desirability factor was chosen as the optimum process parameters. The results presented in Tables 3, 4 and 5 show that 150 °C, 27 bar and 92.13 min were optimum HTC conditions, while 208 °C, 22.5 bar, 331 min and 20.02:4.98(CT + CS:SS) were optimum conditions for the Co-HTC process. The error percentages found are presented in Table 6 and show that the optimized results were consistent compared to the experimental measurement.

**Table 6:** Hydrothermal carbonization and co-hydrothermal carbonization optimum operating conditions.

Name	HCT	HCS	HCB	POAE
**Parameters**				
Temperature (°C)	150	150	208.70	0–0.33
Pressure (bar)	27	27	22.55	0–0.22-
Time (Min)	92.13	10	331.02	0.35–7.4
CT + CS:SS	–	–	20.02:4.98	1.96
**Results (%)**				
Ash content	38.13	21.12	17.43	0.07–1.09
Fixed Carbon	40.20	57.19	58.88	0.5–1.21
Volatile Matter	21.67	21.69	23.69	1.08–0.37
Mass yield	96.18	96.05	84.67	1.35–2.00

*HCT* coal tailing’s hydrochar, *HCS* coal slurry’s hydrochar, *HCB* hydrochar from the Co-HTC of coal and sewage sludge, *SS* sewage sludge, *POE* percentage of absolute error.

The comparison of the conditions used in this work with the HTC and Co-HTC operating conditions of different feedstock utilized in previous show that the Co-HTC process has a significant carbonization potential to produce HCB comparable to HCB obtained from other feedstocks reported in literature^9,12,14^. Furthermore, the conditions selected in this study could reduce the energy need and the cost of the process from the development and economic feasibility perspectives compared to reported operations conditions used for other thermal process such as pyrolysis and gasification^16^.

Post analyses were performed to assess the accuracy of the model using optimum parameters as confirmation points (Table 6). Post experiment results with fixed carbon (FC) of 40.19%, 57.19% and 58.88% content for HCT, HCS and HCB respectively were used to validate the regression models. The predicted means were compared to the medians using the regression models and the standard deviations and standard errors obtained were in acceptable range (< 2%). This further confirms that the models developed were valid and could be used to predict the FC of the produced hydrochar^29^.

### Characterization of the synthesised hydrochar

The proximate analysis results of raw materials and produced hydrochar (HC) from hydrothermal carbonization (HTC) and Co-hydrothermal carbonization (Co-HTC) at optimum conditions are presented in Table 7. The results show that the FC content of the raw samples increased through HTC and Co-HTC process as presented in Table 7. The HTC and Co-HTC process present an opportunity to upgrade the FC content of raw materials through the release of mineral matter, oxygen, and sulphur content from their molecular structures^30^. The HTC and Co-HTC optimum conditions maintained the water at a liquid state and favoured the hydrolysis of aliphatic components, dissolution of minerals matter and prevent the escaping of the carboxylate’s gas. The change in FC of coals-sewage sludge blend (CB) after Co-HTC process was greater compared to the hydrothermal carbonization (HTC) of coal tailing (CT) and coal slurry (CS) individually as shown in Table 7. This may be due to the increase of acidic conditions produced from the decomposition of cellulose, chlorine, and hemicellulose constituents of the sewage sludge into organic acid monomers during decarboxylation and dehydration reactions of Co-HTC process^31^. As a consequence, the lower pH of Co-HTC process liquid increases the sulphur removal (formation of sulphite and sulphate) and allows the inorganic elements to be released into the liquid phase^12,32^. This eventually led to a reduction of the ash content present in the feedstock and increases the FC of the produced hydrochar. However, the increase of temperature above the 150 °C and 208 °C for HTC and Co-HTC respectively (Tables 3, 4 and 5) proportionally increases the ash content of the produced HC due to the condensation of components dissolved in the solid hydrochar and liquefaction of carbon from the feedstock^33^. The results presented in Table 7 show that Co-HTC products had significantly lower ash content than the feedstock used, produced HCT and HCS respectively. The higher decrease in mass yield observed from the HCB compared to the HCT and HCS can be attributed to the increased decomposition of the blend feedstock occurring under Co-HTC conditions^34^. The further increase in temperature severity of the HTC and Co-HTC (respectively above 150 °C and 208 °C) operating conditions leads to a higher degree of thermolytic decomposition, fragmentation, and solubilisation of macromolecules (coals, biomass, etc.) resulting in decreasing mass yield^35^. The high CD:SS mass ratio maintained higher mass yield of the produced hydrochar from Co-HTC process possibly due to the hydrothermal stability of the coal compared to the SS^12^. However, excess of coal leads to agglomeration and stability of mineral matter content in the reaction medium and reduces the formation of acidic mild condition which negatively affects the carbonization yield by decreasing the FC content of the produced HCB (Table 5).

**Table 7:** Physicochemical properties of produced hydrochar.

Analysis	Standards used	CT	HCT	CS	HCS	SS	CB	HCB
**Proximate analysis (wt%, adb)**								
Moisture	ASTDM 5142	4.17	1.07	3.94	1.73	8.12	2.71	1.72
Ash	ASTDM 5142	38.64	37.96	23.22	22.78	36.07	37.75	19.56
Volatile matter	ASTDM 5142	21.44	21.04	21.28	18.38	47.07	21.92	20.31
Fixed carbon	ASTDM 5142	35.75	39.94	50.98	57.11	8.75	37.62	58.41
**Ultimate analysis (wt%, adb)**								
Carbon	ISO 12, 902	42.82	49.8	61.85	66.9	29.7	45.64	67.04
Hydrogen	ISO 12, 902	3.01	2.87	3.56	2.98	4.88	3.05	2.78
Nitrogen	ISO 12, 902	1.14	1.72	1.39	1.99	4.15	1.82	2.43
Oxygen	By difference	8.79	5.83	4.78	2.75	15.22	8.2	6.08
Total sulfur (wt%, adb)	ISO 19,579 : 2006	1.43	0.75	1.26	0.87	1.86	0.83	0.39
Mass yield (%)			82.66		86.74			65.38
**Calorific values (MJ/kg)**		16.59	19.33	24.4	25.79	13.2	17.68	24.31

*CT* coal tailing, *CS* coal slurry, *HCT* hydrochar coal tailing, *HCS* hydrochar coal slurry, *SS* sewage sludge, *CB* blend CT + CS + SS, *HCB* hydrochar from the blend CT + CS + SS, Solid/liquid ratio:1/4, *adb*: air dried basis. Oxygen% = 100- (Moiture + Ash + Total Carbon + Hydrogen + Nitrogen + Sulphur).

The HTC and Co-HTC operating conditions considerably modified the elemental compositions of the HCT, HCS and HCB produced from CT, CS and CB respectively (Table 7). At optimum conditions, much of the atomic carbon content from the feedstock appear to remain retained in the solid HC produced showing the potential of sequestration and repolymerization of HTC and Co-HTC process^36^. The increase in total carbon content in the produced HC corresponds to the decrease in the hydrogen, oxygen, and total sulphur content (Table 7). The decrease of sulphur content in the produced hydrochar, possibly also contributes to the liberalization of pores and improve the textural structure as demonstrated in previous studies^16,37^. The carbon densification factors for the HCT, HCS and HCB, were higher than 1 in all cases and were consistent with the values reported in previous studies on HTC and Co-HTC process and showed the evidence of carbonization^12^. HTC and Co-HTC carbonization yields (Cy) were determined to evaluate the degree of carbonization. The results obtained show that carbonization yields (Cy) of 96.13%, 93.82% and 96.03% for HCT, HCS and HCB respectively which were consistent with previous results on HTC of sub-bituminous coal and Co-HTC of coal and biomass^12^. The results also show that nitrogen increased slightly possibly due to the stagnation of nitrogen under HTC and Co-HTC operating conditions, and the utilization of nitrogen gas inlet to maintain the inert medium. The sulphur content of the HCB (0.39%) was lower compared to the sulphur content of the feedstocks, HTC and HCS respectively, demonstrating the efficiency of Co-HTC process for synergetic sulphur content reduction in coals and biomass. The elemental composition changes implied the structural changes of the produced HCT, HCS and HCB^38^.

**Figure 8:**
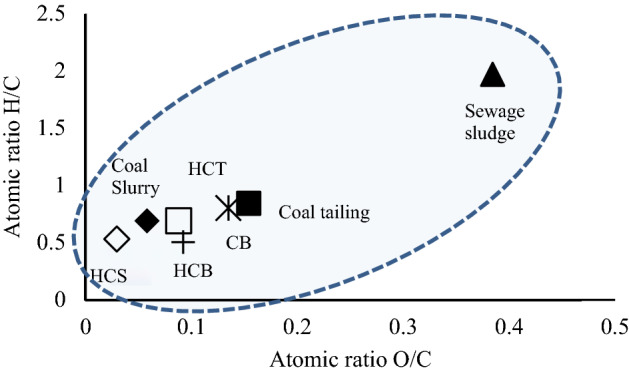
Van-Krevelen diagram for untreated, HTC, and Co-HTC hydrochar.

The atomic ratios of H/C and O/C were determined from elemental compositions (Table 7) and evaluated using the plot of Van Krevelen diagram, as shown in Fig. 8. The atomic ratios plots show that the raw materials shifted from up to down and from the right to the left, demonstrating the progression of carbonisation process and the effects of HTC and Co-HTC operating conditions on the carbonisation degree. The observation of the Fig. 8 implies that the HTC and Co-HTC processes may be driven by a succession of dehydration, and decarboxylation reactions causing the decrease of H/C and O/C atomic ratios respectively. The material representative point closer to the origin indicates a higher carbon content. The CT and CS were found close to the origin compared to the SS, as coal discards have higher carbon content. The produced hydrochar points are lower and further toward the left showing the decrease of hydrogen and oxygen content. Figure 8 further shows that the feedstock possibly undergoes dehydration as primary reaction followed by decarboxylation during HTC and Co-HTC process. The carbon content of the CB increases by 46.88%, while hydrogen and oxygen decrease by 8.85% and 25.85% respectively. This may be attributed to the loss of hydroxyl groups as shown in Fig. 3 possibly supporting the evidence that the dehydration reaction trend for HTC and Co-HTC ending with condensation of carboxylates created during decarboxylation reaction to produce a hydrophobic restructured carbonaceous material^12^ as shown in Fig. 8.

**Figure 9:**
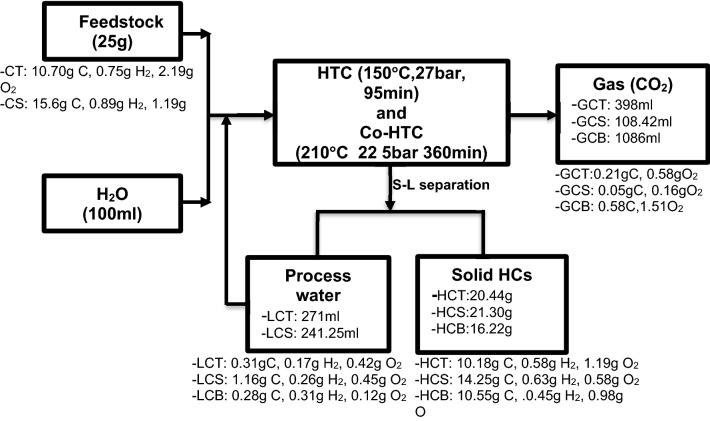
HTC, and Co-HTC process mass balance.

Figure 9 presents the overall experimental mass balance of HTC and Co-HTC for this work. These results show that after thermochemical decomposition of CT, CS and CB, the major carbon content of the feedstock ends in the restructured and condensed HCs (aromatic polymer). This is consistent with previous studies that described the overall HTC products as primarily consisting of acetic acid, methanol, carbon dioxide, and a solid hydrochar^12^, and that the overall thermal decomposition during HTC could be represented by the reaction (1), where the chemical composition of the HTC solid products is estimated in terms of C, H, and O deduced from elemental compositions, as shown in Table 7.

Reaction 1 $$ {\text{C}}_{{\text{x}}} {\text{H}}_{{\text{y}}} {\text{O}}_{{\text{z}}} + {\text{H}}_{{2}} {\text{O}} \to {\text{C}}_{{\text{m}}} {\text{H}}_{{\text{n}}} {\text{O}} + {\text{CH}}_{{3}} {\text{COOH}} + {\text{CH}}_{{3}} {\text{OH}} + {\text{CO}}_{{2}} $$

In this reaction, the solid HC is noted C_m_H_n_O, the liquid phase was assumed to be composed by CH_3_COOH and CH_3_OH produced after the hydrolysis, decarboxylation and demethanation when methane and carboxylates ions reacted in aqueous phase^12,34,36^.

The overall theoretical HTC mass balance represented by the combined reactions (2)–(4):

Reaction 2 $$ {2}.{\text{16C}}_{{7}} {\text{H}}_{{6}} {\text{O}} + {2}.{\text{16H}}_{{2}} {\text{O}} \to {\text{C}}_{{{12}}} {\text{H}}_{{8}} {\text{O}} + 0.{\text{66C}}_{{2}} {\text{H}}_{{4}} {\text{O}}_{{2}} + {1}.{\text{66CH}}_{{3}} {\text{OH}} + 0.{\text{16CO}}_{{2}} $$

Reaction 3 $$ {2}.{\text{1C}}_{{{18}}} {\text{H}}_{{{12}}} {\text{O}} + {3}.{\text{8H}}_{{2}} {\text{O}} \to {\text{C}}_{{{33}}} {\text{H}}_{{{18}}} {\text{O}} + {\text{C}}_{{2}} {\text{H}}_{{4}} {\text{O}}_{{2}} + {2}.{\text{7CH}}_{{3}} {\text{OH}} + 0.{\text{1CO}}_{{2}} $$

Reaction 4 $$ {2}.{\text{6C}}_{{8}} {\text{H}}_{{6}} {\text{O}} + {4}.{\text{8H}}_{{2}} {\text{O}} \to {\text{C}}_{{{15}}} {\text{H}}_{{{14}}} {\text{O}} + {2}.{\text{4C}}_{{2}} {\text{H}}_{{4}} {\text{O}}_{{2}} + 0.{\text{4CH}}_{{3}} {\text{OH}} + 0.{\text{6CO}}_{{2}} $$

**Figure 10:**
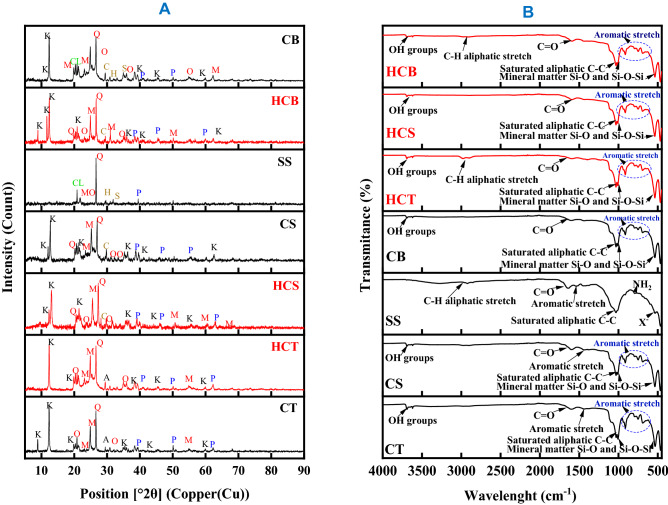
XRD patterns of coal, sewage sludge and produced hydrochar (**A**) and FTIR spectra of coals, sewage sludge and produced hydrochar (**B**).

The diffractogram presented in Fig. 10A shows XRD results of the CT, CS, SS and produced HCs samples which show that CT and CS have broad peaks corresponding to coal patterns rich in amorphous phases while the SS presented a predominant crystal lattice^39^. The amorphous hump observed on the XRD pattern of CS reveals its high content in organic matter compared to CT^39–41^. The mineral phases identified and labelled accordingly on the diffraction patterns presented on Fig. 10A are; Clays: K (Kaolinite); Silicates: M (Muscovite), A (Anorthite), Q (Quartz), MO (Moganite), O (Orthoclase); Carbonates: S (Siderite), C (Calcite); Oxide: H (Hematite); Sulphurs: P (Pyrite), and Chlorite: CL (Clinochlore). The mineral phase identification indicate that silicates ([SiO_(4−x)_]n) and aluminosilicates (KAl_2_(AlSi_3_O_10_)(OH)_2_) (clay) minerals are present in significant proportion in the CT and CS while the predominant mineral phases in the SS are quartz (SiO_2_) and clinochlore ((Mg, Fe(^2+^)5Al_2_Si_3_O_10_(OH)_8_), showing the trigonal, hexagonal, triclinic and monoclinique, crystal systems of the three materials respectively^42^. The diffraction patterns of the mixtures of the three materials CB revealed an interaction between minerals phases resulting in change of structure (orthorhombic) and peaks intensity^43^. The decrease of peaks intensities of mineral phases observed in the diffraction patterns of the produced hydrochar followed the HTC and Co-HTC operating conditions effect on the possible the dissolution of mineral content of the feedstock. Figure 8 also indicate a significant decrease of mineral phases peak’s intensities for Co-HTC process which may be due to the decomposition of SS into organic acid monomers during decarboxylation and dehydration reactions as also shown in Fig. 8. The organic humps become intense and broader around 20° 2θ in the HCB diffractogram (Fig. 10A). Previous works attributed these formations of hump on XRD diffractogram to the progressive transformation of crystalline structure into amorphous structure. That has been corroborated in previous studies which showed that the condensation and polymerisation of carboxyl and carbonyl groups produced from decarboxylation during HTC and Co-HTC forms an amorphous hydrophobic aromatic polymer^44,45^. These may explain the observed residual intense peaks on the HCT and HCS from the CT and CS respectively, showing higher degree of crystallinity compared to HCB.

The FTIR spectra (Fig. 10B) were used to describe the chemical structural properties of the raw materials and produced hydrochar. The peaks at 3620 cm^−1^ band were attributed to O–H stretching vibration linked with kaolinite mineral phase presented on the XRD patterns in the form of silanol (Si–OH). The Si–OH groups are instable due to the polarization in aqueous medium (anionic charge) during HTC and Co-HTC process. Hence, water is eliminated and weakens the Si–O back bonds to generate –Si–H and new Si–OH bonds as seen on Fig. 10B^46^. The lower intensity of the O–H observed on the spectrum band of the produced hydrochar compared to untreated coals demonstrated the evidence of dehydration reactions during HTC and Co-HTC process resulting in the release of hydroxyl groups in liquid phase which form water (Eqs. 1 and 2) and as also suggested by the van krevelen plot (Fig. 8). The intense peak at 2922.63 cm^−1^ band was due to higher presence of aliphatic group in the SS characterized by long linear chains –CH_2_ and –CH_3_ asymmetric and symmetric vibrations(alkanes) respectively^47^. The shoulder peaks at 2960 cm^−1^ band observed on the HCT and HCB spectra were assigned to asymmetric aliphatic –CH_3_ stretching vibration^48^. The increase of peaks intensity of the C–H aliphatic stretch observed at 2850 cm^−1^ and 2920 cm^−1^ from CT and CS spectrums may be due to the interaction between C–H alkyl groups^14,47,48^. The peak observed at 1700 cm^−1^ band was attributed to the aliphatic C=O and –COOH stretching vibrations of carboxyl and carbonyls, mainly ketones, aldehydes, and esters in the SS^47^. At 1600 cm^−1^ bands, corresponding to the aromatic C=C structure, HCS possesses the strongest peak intensity followed by HCB, CS, CB, HCT, CT and SS. It has been argued that a strong transmittance peak at 1600 cm^−1^ wavelength indicates a high carbon content present in the material analysed validating the presence of lignin^49^. This explanation is consistent with the distribution of total carbon content obtained from ultimate analysis of materials presented in Table 7. The peaks observed at 1595.25 cm^−1^ wavelength indicated the presence of double bound carbonyl groups (C=O) combined with aromatic stretch confirming the presence of cellulose^50^. The intensity of C=O peaks of the produced hydrochar was clearly weak compared to the feedstock. This is possibly an indication of the decarboxylation reaction occurring during HTC and Co-HTC process as described by Eq. 3. The peaks at 1391 and 1240 cm^−1^ band were identified in the CT, CS and CB but not in the SS. These peaks were attributed to the symmetric deformation associated with the –CH_3_ groups and phenol^51^. The peaks observed at 1250 cm^−1^ revealed the saturated aliphatic skeletal C–C vibrations^51,52^. The transmittance peak within the fingerprint region of the spectrum at 1030 and 570 cm^−1^ band in all spectra with decreasing intensity from CT, SS, CS, CB, HCS, HCT to HCB were assigned to the vibrations associated with Si–O–Si in quartz and kaolinite^53^. Multiple peaks observed between 900 and 800 cm^−1^ not appearing in the SS were assigned to aromatic species in aromatic rings; trans- and cis-CH_2_ in long saturated aromatic –CH–CH chains, C–O stretching vibration of ether groups; O–H bending vibrations in phenolic, phenoxy and hydroxybenzene structures and were mostly observed with strong intensity in the feedstock compared to produced hydrochar. This may be due to the condensation and polymerization reactions occurring in HTC and Co-HTC processes resulting in elimination of the oxygen and hydrogen into water in liquid phases and restructuration of the carbon skeleton^54^. This is also supported by elemental analyses (Table 7) which show the decrease of oxygen content from the feedstock to the produced hydrochar with HCB is the most oxidized product obtained due to the decomposition of cellulose^49,50^. The peaks at 866 cm^−1^, 805 cm^−1^, and 745 cm^−1^ bands revealed the presence of aromatic nucleus –CH bending vibrations in the coals, the CB, and the HCB^47,51^. Hence, HTC and Co-HTC can be characterized as thermochemical process driven by dehydration (Reaction 5 and 6), decarboxylation (Reaction 7), and demethanation (Reactions 8 and 9)^13^.

Reaction 5 $$ {\text{2R}}{-}{\text{OH}} \to {\text{R}}{-}{\text{O}}{-}{\text{R}} + {\text{H}}_{{2}} {\text{O}} $$

Reaction 6 $$ {\text{2R}} {-} {\text{CH}}_{{2}} - {\text{O}} {-} {\text{CH}}_{{2}} {-} {\text{R}} \to {\text{R}} {-} {\text{CH}} = {\text{CH}} {-} {\text{R}} + {\text{H}}_{{2}} {\text{O}} $$

Reaction 7 $$ {\text{RCOOH}} \to {\text{RH}} + {\text{CO}}_{{2}} $$

Reaction 8 $$ {\text{2R}} {-} {\text{CH}}_{{3}} \to {\text{R}} {-} {\text{CH}}_{{2}} {-} {\text{R}} + {\text{CH}}_{{4}} $$

Reaction 9 $$ {\text{R}} {-} {\text{CH}}_{{2}} {-} {\text{CH}}_{{2}} {-} {\text{CH}}_{{2}} {-} {\text{R}} \to {\text{R}} {-} {\text{CH}} = {\text{CH}} {-} {\text{R}} + {\text{CH}}_{{4}} $$

where R represents the remainder of a cellulose or lignin molecule to which the reacting groups are attached.

**Figure 11:**
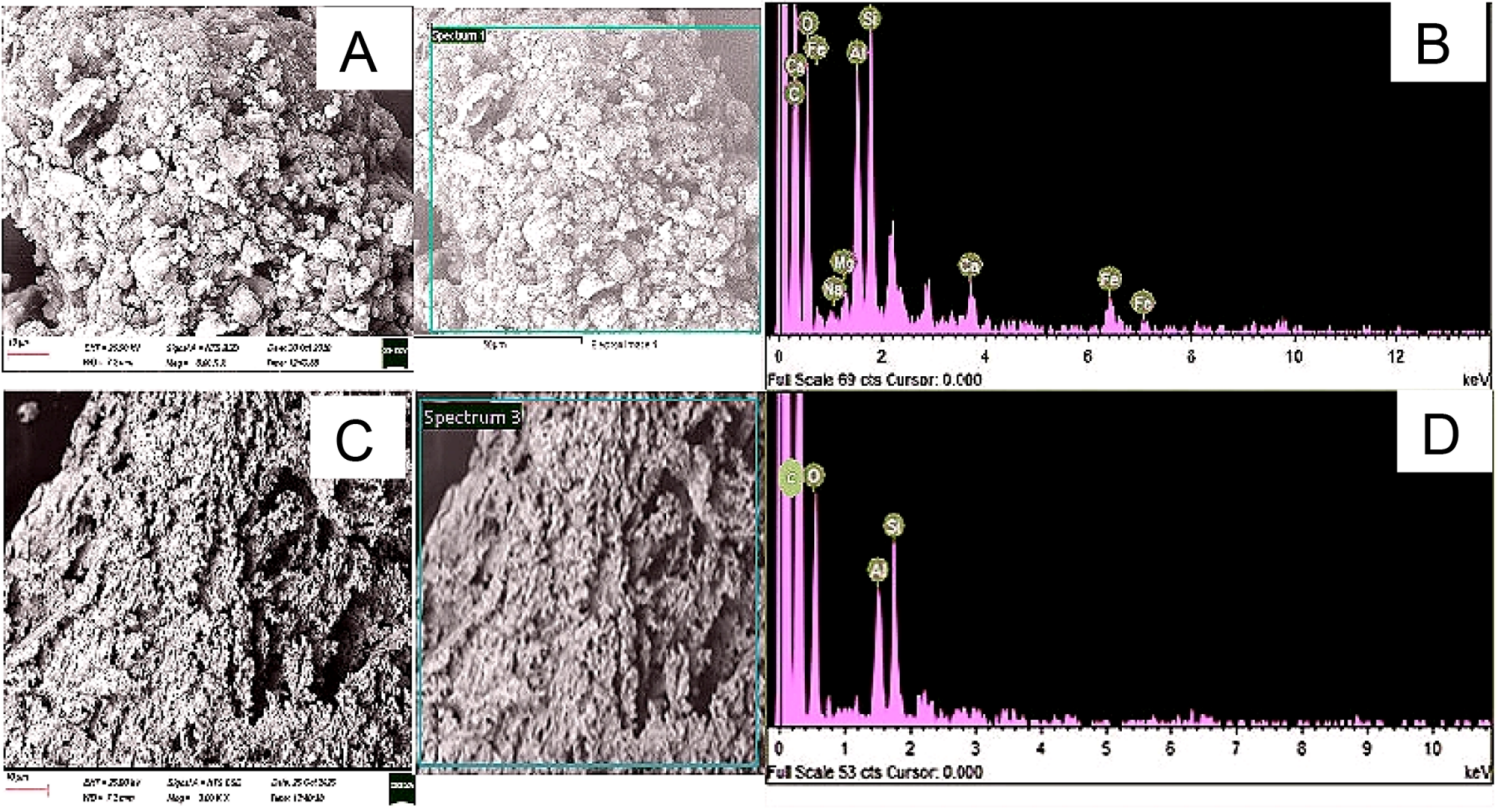
SEM images (**A**,**C**) and EDX elemental spectrums (**B**,**D**) of coal tailing (up), and coal tailing’s hydrochar (down).

**Figure 12:**
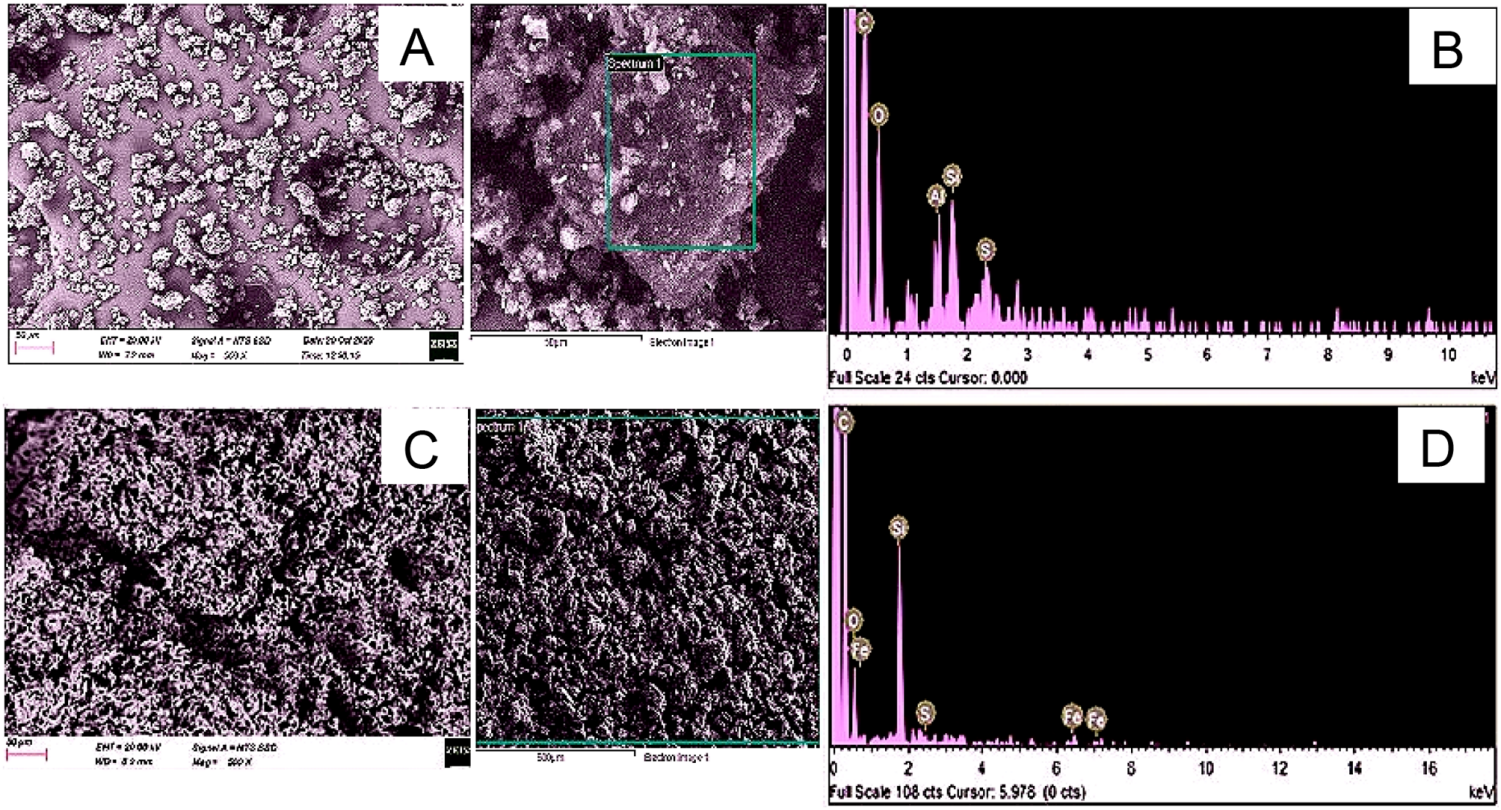
SEM images (**A**,**C**) and EDX elemental spectrums (**B**,**D**) of coal slurry (up), and coal slurry’s hydrochar (down).

**Figure 13:**
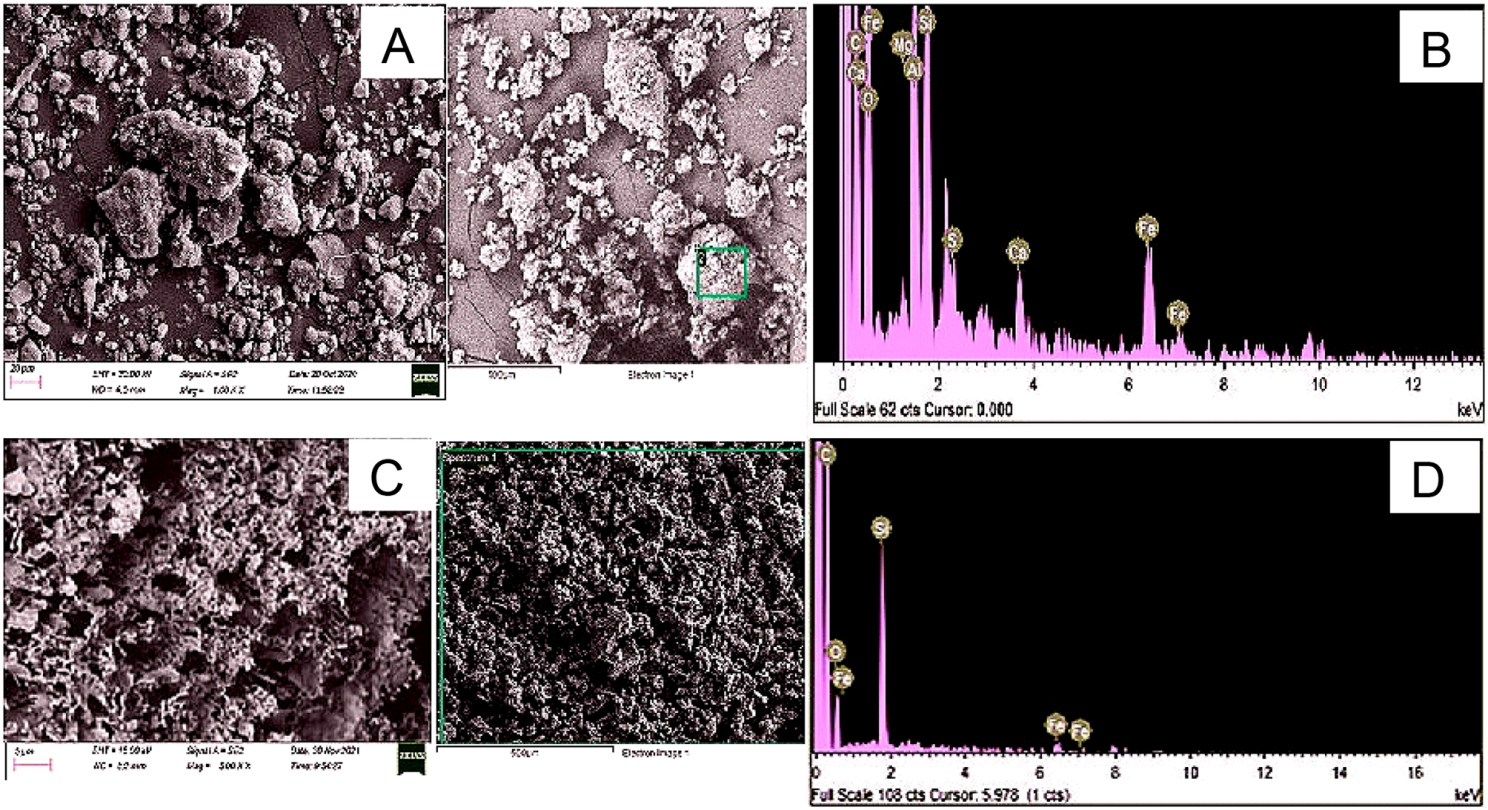
SEM images (**A**,**C**) and EDX elemental spectrums (**B**,**D**) of the mixture of coals and sewage sludge blended in optimized ratio (1CT + 1CS:0.4SS) (up), and the Co-HTC produced hydrochar (HCB) (down).

Additionally, the surface structural arrangement of feedstocks and produced HC were investigated by scanning electron microscopy (SEM). Figures 11, 12 and 13 reveal that the physicochemical properties of the feedstock were vastly impacted by HTC and Co-HTC optimum conditions. The element spectrums of the produced HC displayed the predominance of carbon confirmed by higher peaks. The reduction (Si, O and Fe) and disappearance (Al, Mg and Na) of peaks on the spectrums from the feedstocks to the produced HC were proportional to the decrease of elemental mineral composing the ash content under HTC and Co-HTC conditions^55^. This correlates with the proximate and elemental analyses. The SEM image of CB (Fig. 13a) shows aggregating particles identified as SS on the coal surface, which is supported by the presence of carbon, oxygen, silica, and aluminium peaks on the element spectrum of the selected region. Thus, coal served as a reaction site for sewage sludge particles, which interacted and favoured complete mixing of the feedstock during Co-HTC, resulting in surface homogenisation of the produced hydrochar (Fig. 13c). Furthermore, the SEM images of the feedstock and produced hydrochar revealed a hydrothermal-induced rearrangement from rough and irregular morphology in the raw materials (Figs. 11a, 12a, 13a) to well distributed porous morphology in the produced hydrochar (Figs. 11c, 12c, 13c). Images of the produced hydrochar indicated spotting features and the formation of microstructural pores. These properties provided potential absorbent properties for the hydrochar produced^56^. The thermolytic effect of HTC and Co-HTC temperature increase on the feedstock generates pores in the material structure through the release of hydrogen, oxygen, sulphur, and the degree of mineral content dissolution. This is in accordance with other results that demonstrated that the process severity conditions affect the thermolytic decomposition, devolatilization reaction characteristics, and porous structure formation of HTC and Co-HTC^12,57^. Hydrochar has smoother and more homogeneous surface morphologies than feedstock. The pores size uniformity on the surface images of produced hydrochar show a difference in the degree of mineral dissolution for HTC (Figs. 11a and 12a) and Co-HTC (Fig. 13a) caused by an increase in acidic mild conditions^12,57^. The surface morphology of the produced hydrochar obtained from SEM favoured the evaluation of microstructural changes of the coals and the mixture coal-sewage sludge after HTC (Figs. 11a and 12a) and Co-HTC (Fig. 13a) process respectively.

**Figure 14:**
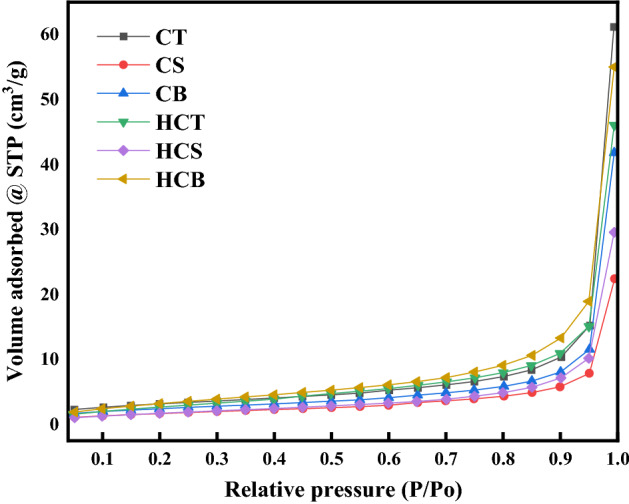
Isothermal adsorption plots of and Co-HTC hydrochar.

The isotherms of nitrogen adsorption of the feedstocks and produced hydrochar are presented in Fig. 14 and were categorized as type III isotherms according to IUPAC isotherm plots classification (lateral interactions between adsorbed molecules are strong in comparison to interactions between the adsorbent surface and adsorbate)^58^. These isotherms show the volume of nitrogen adsorbed at lower P/Po and displayed folds followed by loops revealing the development of mesopores created by the devolatilization and sulphur release under HTC and Co-HTC conditions^59^

The coal slurry’s hydrochar pore diameter was larger than the CT and produced HCT which decreases the nitrogen storage capacity as seen on the limit relative pressure of the associated isotherm loop^60^. The HCB isotherm show that the Co-HTC conditions allowed the SS (biomass) to settle on the coal surface, adsorb, and form a rudimentary layer resulting in a reduced pore diameter obtained (Fig. 14). The aggregation of SS particles observed on SEM images of CB contributes to the formation of narrowed pores of the produced HCB which increases the nitrogen adsorption observed on the associated isotherm from the relative pressure of 0.8 and upward^12,60^. The description of porous materials internal structure is done by the determination of pore size distribution analysis using a simplified model determined as follow^61^.

8 $$f\left(W\right)=\frac{dV}{dW}$$

where W is pore width and V is the pore volume.

The average pore diameters of CD, SS and produced HCs are presented in Table 8. The obtained results show a unimodal distribution referring to the presence of pores in the mesopores region (2 nm > pore diameter < 50 nm)^61^. The average pore diameters of the produced HC confirm (Table 8) the development of mesopores provoked by HTC and Co-HTC consistent with the pores size distribution of hydrochar and biochar materials produced in previous studies^59,61^.

**Table 8:** BET analysis results of the coals, produced HCs and AC.

Material	Surface area (m^2^/g)	Total pore volume (cm^3^/g)	Average pore diameter (nm)
HCT	11.882	0.071	2.62
CS	6.37	0.048	3.34
HCS	14.35	0.094	2.65
CB	6.17	0.09	3.04
HCB	20.35	1.38	2.05

*CT* coal tailing, *CS* coal slurry, *CB* blend of coals and sewage sludge, *HCT* coal tailing’s hydrochar, *HCS* coal slurry’s hydrochar, *HCB* hydrochar form CB, *BET* Brunauer Emmet Teller.

The XRF results presented in Table 9 show the predominance of Silicon (Si), Aluminum (Al), Iron (Fe), Magnesium (Mg), Manganese (Mn) and Calcium (Ca) in the raw materials (CT, CS, SS) analyzed. Thus, in agreement with the XRD analysis which identified the dominant presence of silicates (quartz, muscovite, orthose) and clay or aluminosilicates (kaolinite) minerals followed by pyrite, calcite, hematite, clinochlore and siderite. The decrease of mineral element in the ash of CT, CS and CB after HTC and Co-HTC respectively as shown in Table 9 is proportional to the reduction of mineral phases peaks intensity observed in Fig. 10A. This may be due to the release of mineral element into the HTC and Co-HTC liquid phases^62,63^. Previous studies have shown that in acidic medium silicates mineral dissolve via multi-step process initiates by rapid exchange of cations at the mineral surface, followed by hydrolysis and subsequent detachment of silica and alumina component from the remaining carbon skeleton^64^. This is in agreement with the FTIR results in Fig. 10B where the release of –OH groups was observed on the spectra of the produced hydrochar. As a result, under acidic conditions produced by HTC and Co-HTC, silicates and aluminosilicates minerals are decomposed by metal and silica dissolution, whereas the carbon skeleton accuses a smaller decomposition before condensation and repolymerization^14,61^. Therefore, the decarboxylation that occur during HTC and Co-HTC, lead to a breakdown of C=O and –COOH identified in the feedstock spectra, producing organic acids in the aqueous system. This decreases the pH and subsequently causes a partial dissolution of mineral elements. The lower pH medium produced by the decomposition of extended aliphatic groups combined to double bound carbonyl groups (C=O) present in SS under Co-HTC enhance the dissolution of minerals. As a consequence, the mineral elemental composition in the produced hydrochar decrease significantly (HCB). The analysis of liquid phases from HTC (LCT, LCS) and Co-HTC (LCB) indicate the amount of metal dissolved following each process. Consequently, the XRF results presented in Table 9 indicate a decrease in the major mineral elements present in CB, CT, and CS, respectively. As shown in Table 9, minor elements such as Potassium (K), Sodium (Na), Titanium (Ti), Manganese (Mn), Phosphorus (P), Chromium (Cr), and Nickel (Ni) decreased and in some cases disappeared from the produced hydrochar. The XRF results (Table 9) shows the comparative mineral dissolution evaluation of Co-HTC and HTC. Table 9 shows that the majority of the mineral components identified in the feedstocks were present in relatively stable form in the synthesized HCs, as determined by XRF. Therefore, the adsorption application of synthesized HCs for decontamination of polluted waters or environmental remediation is unlikely to be able to separate the stable element mineral complexes of the HCs back into the environment. The increase in LOI is consistent with previous findings that determined that the increase in LOI in carbonaceous material corresponds to the increase in its organic matter, which contains carbon^65^. The results show that the blended treatment of CT, CS, and SS in Co-HTC results in a higher carbonization yield (%) than HTC of CT and CS alone.

**Table 9:** XRF results of HC products.

Element formula	Element composition	Standard deviation	CT	CS	SS	CB	HCT	HCS	HCB
Conc (%)	Conc (%)	(+/−) ppm							
Si	SiO_2_	2	17.021	6.89	11.43	11.78	14.99	6.72	8.43
Al	Al_2_O_3_	3	7.14	4.48	5.94	5.85	6.29	4.37	4.19
Fe	Fe2O3	1	3.31	1.26	5.97	3.51	2.91	1.23	2.51
Ca	CaO	3	5.1	1.72	2.7	3.17	4.49	1.68	2.27
Mg	MgO	2	0.46	0.23	1.15	0.61	0.41	0.22	0.44
K	K_2_O	2	0.43	0.19	0.85	0.49	0.38	0.19	0.35
Na	NaO_2_	2	0.074	0.059	0.58	0.24	0.07	0.06	0.17
Ti	TiO_2_	1	0.4	0.33	0.51	0.41	0.35	0.32	0.30
Mn	MnO	3	0.023	0.015	0.16	0.07	0.02	0.01	0.05
P	P_2_O_5_	2	0.14	0.15	4.3	1.53	0.12	0.15	1.09
Cr	Cr_2_O_3_	3	0	0	0.088	0.03	0	0	0.021
Ni	NiO	3	0	0	0.031	0.01	0	0	0.007
LOI	LOI	4	39.78	70.21	66.29	58.76	45.18	71.94	76.28

*CT* coal tailing, *CS* coal slurry, *SS* sewage sludge, *CB* blend of coal and sewage sludge, *HCT* hydrochar from coal tailing, *HCS* hydrochar from coal slurry, *HCB* hydrochar from the Blend (SS + CS + CT), C:0.5(CT + CS).

As shown in Table 10, the optimized operating conditions obtained in this study were relatively lower than HTC and Co-HTC operating conditions of various feedstock used in previous studies. The carbon content of the HCs generated in this study was comparable to that of the HCs listed in Table 10. In addition, the results of this study indicate that the Co-HTC process has a significant carbonization potential (high carbon content) to produce hydrochar from the combination of coal discard and sewage sludge (HCB), comparable to HCB produced from other feedstock as reported in previous studies. In addition, the conditions selected in this study have the potential to reduce the energy requirement and cost of the process from a development and economic feasibility standpoint, when compared to the reported operating conditions for other thermal processes, such as pyrolysis and gasification^16^.

**Table 10:** Comparison of hydrochar synthesised in this work with literature values.

Material	HTC conditions		Proximate analysis (wt%)			Ultimate analysis (wt%)					Source
	Temperature (°C)	Time (Min)	Ash*	Fixed carbon*	Volatile matter*	C	H	N	O*	S	
Clarion coal (Cc)	–	–	11.7	54.0	34.3	63.8	4.1	1.5	26	4.6	12
HC	200	30	7.1	54.7	38.2	71.4	4.8	1.8	19	3.0	
HC	230	30	10.4	53.2	36.4	69.2	4.6	1.7	20.8	3.8	
HC	260	30	7.3	57.3	35.4	69.1	4.3	1.7	21.3	3.5	
Miscanthus (Ms)	–	–	2.7	12.8	84.5	48.7	5.7	0.2	45.4	0	
HC	200	30	0.6	15.0	84.4	52.4	5.8	0.3	41.2	0	
HC	230	30	2.5	19.9	77.6	59.3	5.4	0.3	35.0	0	
HC	260	30	0.7	47.8	51.5	69.6	4.4	0.3	25.6	0	
Blend (Cc + Ms)	200	90	4.2	33.1	62.7	58.2	5.3	0.8	34.4	1.2	
HC	230	90	5.8	52.9	41.3	67.2	4.2	1.1	25.6	2.0	
HC	260	90	3.5	53.4	43.1	68.7	4.1	1.1	24.4	1.6	
Sewage sludge (SS)	–	–	56.11	5.32	38.57	48.46	8.2	7.59	34.61	1.14	72
HC	120	30	62.14	4.87	32.99	49.28	8.24	6.63	34.58	1.27	
HC	300	30	79.41	3.42	17.17	57.41	8.74	4.42	28.07	1.36	
Lignite (Ln)			6.22	45.37	48.41	65.70	5.02	0.90	27.84	0.54	
	300	30	6.89	48.61	44.50	68.70	4.78	1.07	25.18	0.27	
Mixture (Ln + SS)	240	30	35.2	28.74	36.06	64.85	5.59	1.94	27.17	0.45	
Bituminous Coal (CW1)	–	–	64.4	15.5	20.1	20.5	1.9	0.5	4.2	9.6	67
HC	230	30	62.8	18.7	18.5	22.5	1.5	0.5	5.4	8.4	
Bituminous Coal (CW2)			67.4	14.2	18.4	18.6	1.8	0.6	4.1	8.5	
HC	230	30	65.4	17.6	17.0	20.2	1.6	0.7	5.2	7.9	
Food waste (FW)			9.6	18.8	71.6	39.3	6.0	1.5	44.0	0	
HC	280	30	5.1	39.4	55.5	71.7	5.9	4.6	12.8	0	
CW1 + FW	230	30	31.3	29.1	39.6	46.7	2.3	1.0	17.2	2.1	
CW2 + FW	230	30	28.6	31.2	40.2	49.5	4.5	1.9	14.8	1.4	
Sub-bituminous coal (SbC)	–	–	25.5	40.5	34	75.1	5.1	1.1	18.4	0.33	73
Food waste	–	–	6.41	14.48	79.11	39	7.32	5.7	47.68	0.2	74
SbC + FW	300	60	2.21	47.43	50.36	73	7.01	5.17	17.09	0.2	
Sewage sludge	–	–	28.6	1.42	69.98	36.7	6.4	5.5	35.9	9.5	
HC	200	360	43.89	5.47	50.64	33.3	4.4	2.1	18.5	3.8	
Coal			11.74	55.48	32.78	64.07	4.24	1.23	18.07	0.65	75
HC	340	60	7.16	65.12	27.72	74.81	3.5	1.88	12.15	0.56	
Coal discard (CD)	–	–	41.95	35.83	20.17	48.90	2.67	1.15	1.93	1.34	76
Searsia lancea Trees Grown (Ssl)	–	–	3.89	18.41	69.40	45.12	6.35	0.44	35.78	< 0.10	
HC (CD + Ssl)	280	30	1.49	50.33	46.12	–	–	–	–	–	
Coal tailing (CT)	–	–	40.32	37.31	22.37	42.82	3.01	1.14	12.96	1.43	This study
HC	150	95	38.36	40.37	21.27	49.80	2.87	1.72	6.9	0.75	
Coal slurry (CS)			24.17	53.02	22.69	61.85	3.56	1.39	8.72	1.26	
HC	150	95	20.12	57.69	22.19	66.90	2.98	1.99	4.48	0.87	
Sewage sludge (SS)	–	–	39.25	9.52	51.23	29.7	4.88	4.15	23.34	1.86	
CT + CS + SS blend	–	–	38.8	38.67	22.53	45.64	3.05	1.82	10.91	0.83	
HC	208.10	360	19.71	58.82	21.46	67.04	2.78	2.43	7.8	0.39	

*Ash* ash content, *FC* fixed carbon, *VM* volatile matter, *HC* hydrochar.

*Dried basis (moisture free). Oxygen% = 100-(Moiture + Ash + Total Carbon + Hydrogen + Nitrogen + Sulphur).

### Characterization of the HTC process water

The concentration of inorganic elements in produced PW provides information about its treatment prior to discharge or use. The chemical oxygen demand (COD) is the amount of oxygen required to completely oxidize organic carbon to CO_2_ and H_2_O^66^. The COD is an important water quality parameter because, like the BOD (Biological Oxygen Demand), it provides an index to assess the impact of discharged wastewater on the receiving environment^20,66^. Higher COD levels indicate a greater amount of oxidizable organic material in the sample, which reduces dissolved oxygen (DO) levels. A decrease in DO can cause anaerobic conditions, which are harmful to higher aquatic life forms^67,68^. According to a recent study, the produced PW from HTC of different feedstock had a relatively low concentration of inorganic and organic elements when compared to the standard for environmental disposal^69^.

**Figure 15:**
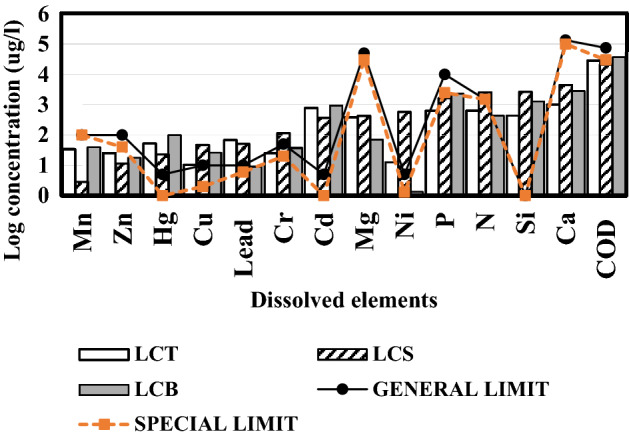
ICP-OES analysis results of LCT: HTC liquid phase from Coal tailing; LCS: HTC liquid phase from coal slurry and LCB: HTC liquid phase from the blending of coal and sewage sludge.

The results presented in Fig. 15 show different element dissolved in the liquid phases after HTC and Co-HTC process. The most abundant elements found in the produced PW were calcium (Ca) > silicon (Si) > nitrogen(N) > phosphorous (P) > Nickel (Ni) > Magnesium (Mg) > cadmium (Cd) > chromium (Cr) > Manganese (Mn). The other elements such as zinc (Zn), copper (Cu), and mercury (Hg) were at lower levels. The absence of the iron (Fe) and sodium (Na) in the analyzed PW indicates that all the Fe and Na content of the raw materials have been retained in the produced hydrochar^70^. The high concentrations of inorganic contaminants and lower pH was observed from the produced LCB compared to LCT and LCS. Thus, confirmed the increased degree of mineral dissolution during Co-HTC due to the decomposition of sewage sludge which produced acidic medium^31^. In addition, according to the standard for the discharge of the PW into the fresh waterbodies, the concentrations of Nitrogen (N), phosphorous (P), cadmium (Cd), chromium (Cr), mercury (Hg), Zinc (Zn), Nickel (Ni) and silicon (Si) approached or exceed the legal limits^71^.The concentration of organic matter in the produced PW revealed the decomposition of organic elements from feedstock under HTC and Co-HTC conditions^49,50^. The concentration of organic matter in the LCB illustrated the complexity of thermal decomposition reactions, interactions between coals and SS which resulted in the fragmentation, and solubilization of carbon macromolecules^50^. The results presented in Fig. 15 show that COD of the produced LCB estimated from organic matter obtained by ICP-OES analysis exceeds the special limit (special limit 30 mg/l) for discharge to fresh waterbodies. The general and special standard limit of elements dissolved in water refer to the maximum concentrations of the elements stated in water used for irrigation and aquatic discharge respectively^72^.

#### Identification of organic compounds

The GC–MS results depicted in Table 11 show that the produced PW from HTC and Co-HTC process contain a variety of dissolved organic compounds, such as organic acids, sugars, phenols etc. The organic compounds are produced from dehydration, decarboxylation and demethylation reactions occurring under hydrothermal process conditions^31^. These compounds are typical of decomposed biomass in the natural environment, but high concentrations in discharge water can pollute receiving waterbodies^70,73^. From the results in Table 11, the concentration of oxime methoxy-phenyl was found higher followed by ascorbic acid 2,6-dihexadecanoate-tricyclo (3.2.2.0) nonan-2-yl 4-nitrophenyl carbonate and silane-(4-bromophenyl) triphenyl. The higher concentration of these component was observed in the LCB compared to LCT and LCS respectively. The results agreed with the concentration of organic matter determined previously from ICP-OES analysis. Thus, confirmed the increased decomposition of coal and sewage under Co-HTC^74^. The concentrations of organic elements dissolved in the produced PW indicate the significancy of its dilution before discharge in waterbodies^75^. However, the produced PW could be recycled to avoid generation of additional wastes. In addition, previous works demonstrated that the use of PW in HTC and Co-HTC increases the carbonization mass yield due to their acidic properties and the condensation of aliphatic fragments content dissolved during precedent hydrothermal process^62^. The acidity of hydrothermal reaction medium strengthens polymerization of benzyl alcohol structure of raw material via the carbonium ion which is the intermediate of the reaction^74,75^. The chemical identification of dissolved organic compound reveals the predominance of carboxylic acid, aromatic compound and low aliphatic hydrocarbons in the produced PW as described in Table 11.

**Table 11:** Identification and concentration of organic compounds in PW.

PW	Retention time (minutes)	Organic compound name	Concentration (%)
LCB	4.055	Oxime-, methoxy-phenyl-	44.85
	4.447	3-pyridinecarboxamide, 6-amino-5-cyano-1,2-dihydro-2-oxo-	3.29
	5.564	Cyclotrisiloxane, hexamethyl-(CAS)	10.07
	11.696	l-(+)-Ascorbic acid 2,6-dihexadecanoate	15.19
	12.493	2-Oxepanone, 7-hexyl-	6.28
	13.46	Beta. Alanine, *N*-methyl-*N*-(1-methyl-4-nitro-1*H*-imidazol-5-yl)-, methyl ester	3.64
	14.657	Hexadecanoic acid, 2-hydroxy-1-(hydroxymethyl)ethyl ester	3.35
	18.412	1-(2-Chloroethyl) -4-dimethylamino-6-methoxy-1,3,5-triazin-2(1*H*)-one	6.34
	19.323	JWH 398N-(4-hydroxypentyl) metabolite	3.74
	22.639	3-{[(2-CHLOROETHYL) (METHYL)AMINO] METHYL}-1,2-DIHYDROXYANTHRA-9,10-QUINONE	3.26
LCT	4.061	Oxime-, methoxy-phenyl-	25.44
	5.306	Tricyclo[3.2.2.0]nonan-2-yl 4-nitrophenyl carbonate	12.85
	6.247	Silane, methyltripropoxy-	4.86
	8.376	6-tert-Butylthiochroman-3-carboxylic acid	5.24
	11.699	Pentadecanoic acid (CAS)	9.94
	12.494	2-Oxepanone, 7-hexyl-(CAS)	6.44
	13.897	2,2,9,9-tetrachloro-3,8-dioxo-4,7-dithia-10,12,12-trimethylbicyclo [8.3.1] tetradec-1(14)-ene	5.56
	14.46	*N*-(3-Dimethylaminopropyl) indole	9.58
	14.66	Hexadecanoic acid, 2-hydroxy-1-(hydroxymethyl)ethyl ester	13.66
	22.62	METHYL 10D-HYDROXYOCTADECANOATE	6.43
LCS	4.006	Oxime-, methoxy-phenyl-	26.77
	4.224	Silane, (4-bromophenyl) triphenyl-	9.54
	6.45	3-(2-Oxooxolan-3-yl) propanoic acid	9.37
	10.2	1,3,4-Thiadiazole, 2,3-dihydro-2-(2-naphthyl)-5-phenyl-	6.84
	10.632	. Beta. -l-Idopyranoside, phenylmethyl 2,4-bis(acetylamino)-2,4,6-trideoxy-, 3-acetate	7.72
	12.407	9-Octadecenoic acid (*Z*)-, methyl ester	7.95
	12.496	2-Oxepanone, 7-hexyl-	8.1
	13.07	(4,6-DICHLORO- [1,3,5] TRIAZIN-2-YL) -(2,6-DIMETHYL-PHENYL)-AMINE	7.29
	14.659	Hexadecanoic acid, 2-hydroxy-1-(hydroxymethyl)ethyl ester	9.72
	15.554	3,5-Dichloro-2-hydroxybenzenesulfonyl chloride	6.7

*PW* process water from HTC and Co-HTC process, *LCT* PW from coal tailing’s HTC, *LCS* PW from coal slurry’s HTC, *LB* PW from Co-HTC of coal and sewage sludge.

#### Determination of total carbon TC and total organic carbon TOC of the produced PW

The results in Table 12 show the overall levels of organic compounds present without a direct correlation between TOC and the total concentration of organic compounds present in the produced PW. However, the TOC composition illustrates the general organic contamination of the PW^32^. The results show that TOC weight percentages are lower than the TIC weight percentage due to the release of organic carbon in the liquid phase during the hydrothermal process^50^. The total organic carbon property of water gives an insight of the amount of oxygen necessary to oxidize all the organic carbon completely^66^. The TOC of LCT and LCB exceeding the special limit for discharge in rivers (11.5 mg/l) of the SA national water discharge regulations creates aerobic conditions which threats aquatic lives^68^. The results in Table 12 show that the produced TOC of PW exceed the special limit of discharge in rivers. This is consistent with the COD results obtained from organic matter determined by ICP-OES analysis. Hence, prior treatment of PW is necessary before its envisioned potential discharge in rivers. A combination of dilution, wet oxidation for the degradation of organic components into CO_2_ and water, and adsorption for the removal of the residual mineral elements could be employed to treat produced PW prior to its discharge in water bodies^68,72,75^. Koechermann et al.^73^ reported that recirculating PW in the HTC process increases the carbonization yield of the produced HCs. Therefore, from an economic and environmental aspect, the PW generated by the HTC and Co-HTC processes was recycled in this study, as indicated in the methodology (Fig. 2).

**Table 12:** Total carbon and total organic carbon.

PW	TC (W %)	TIC (W %)	TOC (W %)
			TOC = TC − TIC
LCT	25.69	13.34	12.71
LCS	23.62	12.83	10.79
LB	26.05	12.51	13.18

*PW* Process water from HTC and Co-HTC process, *LCT* PW from coal tailing’s HTC. LCS: PW from coal slurry’s HTC, *LCB* PW from Co-HTC of coal and sewage sludge, *TOC*, total organic carbon, *TC* total carbon, *TIC* total inorganic carbon.

## Conclusion

This study optimized the hydrothermal carbonisation (HTC) of coal discards from coal beneficiation operations and/or sewage sludge by optimizing a number of process parameters, including biomass type, temperature, and residence time, in order to produce hydrochar with improved physicochemical properties.

The optimized HTC conditions (150 °C, 27 bar, 95 min) for individual CT and CS increased the amount of fixed carbon from 37.31% and 53.02–40.31% and 57.69%, respectively, on a dry basis. As a result, the total carbon content of the coal discards increased from 42.82 and 61.85% to 49.80 and 66.90% in the HCT and HCS produced, respectively. In addition, the ash content of coal discards reported on a dry basis decreased from 40.32 and 24.17% to 38.3 and 20.17% in the HCT and HCS produced under optimized HTC conditions. While the optimized Co-HTC conditions of 208 °C, 22.5bars, and 360 min for a CT + CS:SS blend ratio of 5:1 increased the fixed carbon reported on a dry basis and total carbon content of CB (a blend of CT + CS + SS) from 38.67% and 45.64%, respectively, to 58.82% and 67.0%, respectively. The HCB ash concentration decreased from 38.8 to 19.71%. The carbonization yields (Cy) for HCT, HCS, and HCB were determined to be 113.58%, 102.42%, and 129.88%, respectively. Co-carbonization HTC's yield (Cy) was higher than that of HTC alone, possibly as a result of an increase in acidic conditions caused by the degradation of cellulose, chlorine, and hemicellulose into organic acid monomers during decarboxylation and dehydration reactions of the Co-HTC process. As a result, the lower pH of the Co-HTC process liquid increases the sulphur removal (production of sulphite and sulphate) from 0.83 to 0.39% and possibly allows the release of inorganic elements into the liquid phase. This demonstrates the effectiveness of the Co-HTC process for reducing the sulphur content of waste coals and sewage sludge. HCB synthesized from Co-HTC had comparable physicochemical properties to HCS synthesized from HTC, with the highest carbon content and the lowest ash content (CS). This demonstrated the viability of combining coal waste and sewage sludge to produce high-quality carbonaceous material for a variety of applications.

## Data Availability

All data relevant to the study are included in the article. In addition, the datasets used and/or analyzed during the current study are available from the corresponding author on reasonable request.
